# Anticoagulant versus antiplatelet treatment for secondary stroke prevention in patients with active cancer

**DOI:** 10.3389/fneur.2025.1530775

**Published:** 2025-09-16

**Authors:** Moritz C. Kielkopf, Jayan Göcmen, Selina B. Venzin, Fabienne Steinauer, Mattia Branca, Anna Boronylo, Martina B. Göldlin, Johannes Kaesmacher, Adnan Mujanovic, Gianluca Costamagna, Thomas R. Meinel, David J. Seiffge, Philipp Bücke, Mirjam R. Heldner, Ava L. Liberman, Hooman Kamel, Urs Fischer, Marcel Arnold, Thomas Pabst, Martin D. Berger, Simon Jung, Adrian Scutelnic, Babak B. Navi, Morin Beyeler

**Affiliations:** ^1^Department of Neurology, Inselspital, Bern University Hospital, University of Bern, Bern, Switzerland; ^2^CTU Bern, Institute of Social and Preventive Medicine, University of Bern, Bern, Switzerland; ^3^Institute for Diagnostic and Interventional Neuroradiology, Inselspital, Bern University Hospital, University of Bern, Bern, Switzerland; ^4^Department of Clinical Neurosciences, Stroke Center, Neurology Service, Lausanne University Hospital and University of Lausanne, Lausanne, Switzerland; ^5^Department of Pathophysiology and Transplantation (DEPT), Dino Ferrari Centre, University of Milan, Milan, Italy; ^6^Clinical and Translational Neuroscience Unit, Department of Neurology, Feil Family Brain and Mind Research Institute, Weill Cornell Medicine, New York, NY, United States; ^7^Department of Medical Oncology, Inselspital, Bern University Hospital, University of Bern, Bern, Switzerland; ^8^Graduate School for Health Sciences, University of Bern, Bern, Switzerland

**Keywords:** acute ischemic stroke, secondary prevention, antithrombotic drugs, cancer, embolic stroke of unknown source (ESUS)

## Abstract

**Background:**

Approximately 5–10% of patients with acute ischemic stroke (AIS) have known active cancer. These patients are at high risk for both recurrent AIS and major bleeding. The optimal antithrombotic strategy for cancer-related stroke is uncertain. This study compared clinical outcomes among patients with cancer-related stroke treated with anticoagulant versus antiplatelet therapy for secondary prevention.

**Methods:**

We identified consecutive patients with AIS and active cancer hospitalized at our comprehensive stroke center from 2015 through 2020. Patients with cardioembolic mechanisms were excluded. We used Cox regression and inverse probability of treatment weighting (IPTW) analyses to evaluate the associations between type of antithrombotic therapy at discharge (anticoagulant versus antiplatelet therapy) and the main outcomes of 1-year mortality and long-term recurrent AIS.

**Results:**

Among 5,012 AIS patients, 306 had active cancer. After applying study eligibility criteria, we analyzed 135 patients (median age 72 years; 44% women), of whom 58 (43%) were treated with anticoagulant and 77 (57%) with antiplatelet therapy. The median follow-up time was 495 days (IQR, 57–1,029). Patients treated with anticoagulants, compared to patients treated with antiplatelet therapy, were younger (median 69 versus 75 years), had more metastatic disease (72% versus 41%), and higher median baseline D-dimer levels (median 8,536 μg/L versus 1,010 μg/L). Anticoagulant versus antiplatelet therapy was associated with similar risks of 1-year mortality (adjusted hazard ratio [aHR], 0.76; 95% confidence interval [CI], 0.36–1.63) and long-term recurrent AIS (aHR 0.49; 95% CI 0.08–2.83). The IPTW analyses for 1-year mortality confirmed the results of the main analyses (HR 0.82; 95%CI: 0.39–1.72, *p* = 0.61).

**Conclusion:**

Factors associated with anticoagulant use in patients with cancer-related stroke include younger age, more advanced cancer, and elevated D-dimer. Similar outcomes were seen with anticoagulant versus antiplatelet therapy in these patients highlighting the need for future randomized trials to determine the preferred antithrombotic strategy.

## Introduction

Acute ischemic stroke (AIS) and cancer are major causes of morbidity and mortality. Worldwide, approximately 40% of people are expected to develop cancer in their lifetime, while approximately 25% will suffer an AIS (1). Active cancer is a comorbid condition in 5–10% of patients with AIS, and this stroke subgroup is often referred to as “cancer-related stroke” (2).

Paraneoplastic coagulopathy is often implicated in cancer-related stroke (3). This prothrombotic process is multifactorial and involves platelet, coagulation, and endothelium activation; circulating cancer- and platelet-derived extracellular vesicles; and increased neutrophil extracellular trap formation (4). Patients with cancer-related stroke face an increased risk of more severe strokes, AIS recurrence, and mortality compared to other AIS patients (5).

There are no clear guideline-based recommendations for the preferred antithrombotic strategy in cancer-related stroke. According to the American Heart Association guidelines, further research should be conducted to evaluate the benefit of anticoagulation in persons with stroke attributed to cancer-related hypercoagulability as robust data is lacking (6). In clinical practice, anticoagulants are sometimes empirically administered to patients with cancer-related stroke to treat presumed paraneoplastic coagulopathy (7). However, there are few data to support these practices apart from small biomarker-focused studies without comparator antiplatelet arms (8). Secondary analyses of the NAVIGATE ESUS and ARCADIA randomized controlled trials have demonstrated neutral results in recurrent stroke risk between the anticoagulant and antiplatelet treatment groups (9, 10). However, because both studies included patients with both active and inactive cancer, with the exact proportions of each uncertain, these data should be interpreted with caution.

Given the lack of dedicated, adequately powered clinical trials assessing the most appropriate secondary prevention strategy for cancer-related stroke, more retrospective data are needed. Therefore, we investigated the short- and long-term outcomes of patients with active cancer and AIS stratified by the employed antithrombotic treatment strategy (anticoagulant versus antiplatelet therapy) at discharge in a large institutional registry.

## Methods

### Design

We conducted a retrospective cohort study of consecutive patients treated for AIS at a comprehensive stroke center in Bern, Switzerland between January 1, 2015 and December 31, 2020. These patients were prospectively enrolled in the Swiss Stroke Registry, which was accessed for data analysis.

The study was approved by the local ethics committee in accordance with Swiss regulations (project ID: 2022-01560; Kantonale Ethikkommission Bern), and the requirements for written consent was waived. Access to the study data can be requested from the corresponding author and is subject to clearance by the local ethics committee. This analysis adhered to the STROBE checklist guidelines for cohort studies.

### Population

The study population consisted of patients with AIS and known active cancer at the time of AIS or new cancer diagnosed during the hospitalization for the index AIS. Active cancer was defined according to the criteria recommended by the International Society on Thrombosis and Haemostasis (11). This comprised a new or recurrent cancer that was diagnosed or treated within six months prior to the index AIS, or known metastatic cancer. Hematological malignancies that were not in complete remission for more than 5 years were also considered active. Patients diagnosed with a new cancer during the index hospitalization were considered to have occult cancer at the time of AIS, and were included in the known active cancer group for analyses (12, 13). We excluded patients who were diagnosed with cancer after hospital discharge for the index AIS, as this sequence of events may have influenced antithrombotic management decisions and clinical outcomes.

We excluded patients with focal non-melanoma skin cancer because of their low risk of dissemination as well as patients with breast cancer in complete remission who were receiving maintenance hormonal therapy (11, 14, 15). Other exclusion criteria were (i) death during the index hospitalization or missing follow-up data regarding vital status, (ii) no antithrombotic therapy prescription at discharge, and (iii) a cardioembolic stroke mechanism at discharge necessitating anticoagulation (e.g., atrial fibrillation, mechanical valve). Patients with a determined stroke mechanism typically treated with antiplatelet therapy, such as large artery atherosclerosis, were not excluded because these patients may still have cancer-mediated hypercoagulability and could preferentially benefit from anticoagulant therapy.

### Measurements

Demographic and clinical characteristic data were collected from the Swiss Stroke Registry and electronic health records. These included age at admission, sex, pre-stroke functional status (independency defined as a pre-stroke modified Rankin Scale [mRS] score ≤2), baseline imaging modality, and history of cardiovascular risk factors (prior stroke, hypertension, diabetes mellitus, hyperlipidemia, smoking history, and atrial fibrillation). The presence of multi-territory brain infarcts (involving at least two different cerebrovascular territories) was determined using data from baseline neuroradiological imaging. Data on the primary cancer type, stage (16), and presence of metastasis at the time of AIS was collected from electronic health records. Laboratory measurements included D-dimer, C-reactive protein (CRP), hemoglobin, platelet count, fibrinogen, and lactate dehydrogenase (LDH). For patients with multiple measurements during the index AIS hospitalization, the baseline value was recorded. Two neurologists determined the AIS mechanism at discharge, classified according to the Trial of Org 10,172 in Acute Stroke Treatment (TOAST) criteria and the embolic stroke of undetermined source (ESUS) classification (17, 18). Recent data have indicated that among patients with AIS, there is an inverse association between the presence of a right-to-left shunt (patent foramen ovale [PFO] or atrial septal defect [ASD]) and the presence of cancer, suggesting that paradoxical embolism is not a major cause of AIS in patients with cancer (19). Therefore, we classified patients with PFO/ASD and no other apparent cause of AIS as “undetermined stroke etiology” and, if meeting criteria, as ESUS. The diagnosis of venous thromboembolism (VTE), including deep vein thrombosis and/or pulmonary embolism, in the year before and after the index AIS was recorded.

The study exposure was the type of antithrombotic therapy (anticoagulant or antiplatelet therapy) prescribed at hospital discharge from the index AIS. The anticoagulant group consisted of patients treated with therapeutic doses of any oral or parenteral anticoagulant. These included vitamin K antagonists, low-molecular-weight heparins (LMWH) such as enoxaparin or tinzaparin, and direct oral anticoagulants (DOACs) such as edoxaban, rivaroxaban, dabigatran, or apixaban. Patients treated with recommended dosing regimens adjusted for age, weight, or renal function were classified as receiving therapeutic anticoagulation. The antiplatelet group included standard-dose aspirin, clopidogrel, or both. Patients treated with both therapeutic-dose anticoagulant and antiplatelet therapy were included in the anticoagulant group. The treatment decision was left to the treating physician. According to our internal stroke guidelines, if D-dimer levels at baseline exceed 3,000 μg/L and cancer is present, paraneoplastic coagulopathy should be considered as AIS etiology. When a paraneoplastic coagulopathy is suspected, anticoagulation with LMWH at therapeutic dosage (twice daily) or oral anticoagulants should be considered. As tolerance to LMWH is known to be low in cancer-related strokes (in opposition to patients with VTE), we reported continuation of LMWH prescribed at discharge versus regimen change for secondary prevention at the 3-month clinical follow-up (20, 21).

The primary study outcome was mortality at 1 year after the index AIS. To determine patients’ vital status, we analyzed data from the Swiss Population Registry, which records the vital status of Swiss residents on a monthly basis. Secondary outcomes were (i) a good functional outcome at 90 days, defined as a mRS score of ≤2, (ii) all-cause long-term mortality, (iii) recurrent AIS at 90 days and during the entire follow-up period (iv) symptomatic intracranial hemorrhage (sICH) at 90 days and during the entire follow-up period, and (v) major bleeding besides intracranial hemorrhage or clinically relevant non-major bleeding during the entire follow-up period. The criteria used to define the composite bleeding outcome is provided in the Supplemental methods. For long-term mortality, follow-up time was defined as the time from the index AIS to the date of death for deceased patients or to the last update of the Swiss Population Registry for surviving patients. For incident cerebrovascular events (recurrent AIS or sICH), follow-up time was defined as the time from index AIS to the date of event or to the last documented follow-up in the electronic health record if no event was reported. For 90 days follow-up, only patients with available clinical or telephone follow-up were assessed. The mRS score and incident cerebrovascular events at 90 days were determined through telephone survey or clinical visit by investigators certified in mRS assessment. Symptomatic ICH was determined based on the ECASS III definition (22).

### Analysis

Baseline characteristics were reported as median and interquartile range (IQR) for continuous variables and frequency (percentage) for categorical variables. Differences between groups were assessed using Fisher’s exact test for categorical variables and the Wilcoxon rank-sum test for continuous variables. Kaplan–Meier curves were used to estimate the cumulative rates of time-to-event endpoints. The log-rank test and multivariable Cox regression were used to compare the outcomes between antithrombotic treatment groups. All multivariable models were adjusted for patient age, sex, initial D-dimer level, documented metastases at the time of the index AIS, and the presence of multi-territory brain infarcts. These covariates were selected because they either significantly differed between study groups or they were previously associated with adverse clinical outcomes in patients with cancer-related stroke (5). Adjusted hazard ratios (aHRs) were reported with their associated 95% confidence interval (CI). To investigate the association between treatment groups and recurrent cerebrovascular events (AIS or sICH), additional analyses using mortality as a competing risk were performed (see Supplementary methods). Patients who received intravenous thrombolysis before D-dimer assessment were excluded from the multivariable analyses because intravenous thrombolysis can influence levels of coagulation parameters such as D-dimer and fibrinogen (23).

Subgroup analyses were performed in (1) patients whose index AIS mechanism was cryptogenic and who met ESUS criteria. (2) patients with ESUS and an elevated D-dimer level (defined as a value above the median for the cohort), and (3) patients with ESUS and multiterritory infarcts.

In sensitivity analyses, we excluded (1) patients with combined antiplatelet and anticoagulant therapy and (2) patients with a history of VTE treated with anticoagulant therapy at discharge for the index AIS. Because antithrombotic prescription patterns likely varied according to physicians’ perceptions of how prothrombotic and high-risk individual patients were, we performed a second set of analyses calculating propensity scores and using the Inverse Probability of Treatment Weighting (IPTW) method with stabilized weights to minimize potential confounding (24). For IPTW, we reported hazard ratios (HRs) with their associated 95% CI.

Continuous variables with skewed distributions were logarithmically transformed. Missing data were not imputed. Statistical significance was defined as a *p*-value of <0.05. All analyses were performed using Stata 16 (StataCorp LLC) and R (version 3.6.0, R Core Team).

## Results

### Patient characteristics

**Figure 1:**
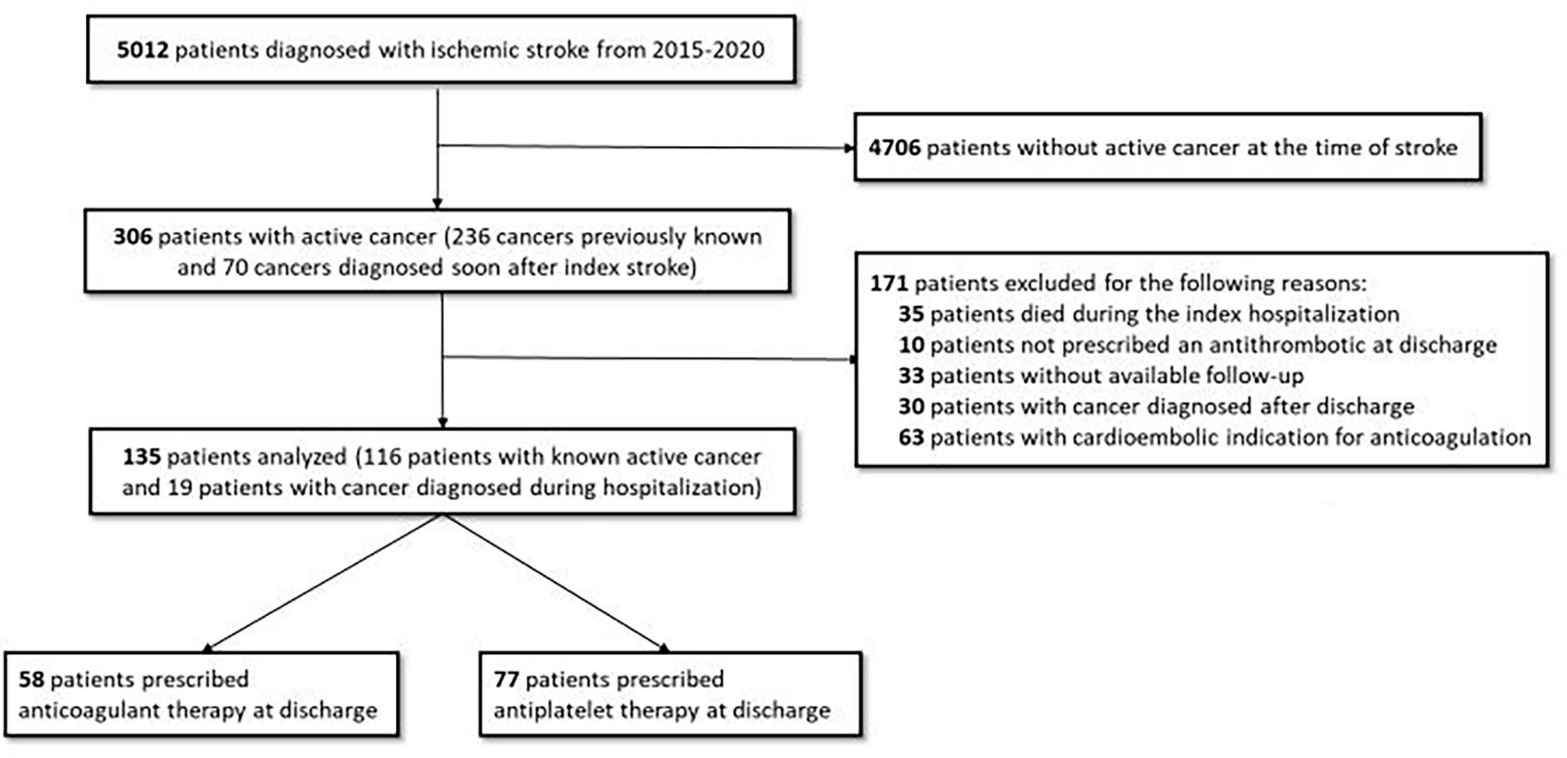
Study flowchart with inclusion and exclusion of patients.

Of 5,012 patients with AIS assessed for eligibility, 306 had active cancer at the time of the index hospitalization (Figure 1 – study flowchart). Among these patients, we excluded 35 who died during the hospitalization, 30 whose cancer was diagnosed after hospital discharge, 33 without available follow-up, 63 with a cardioembolic indication for anticoagulation, and 10 who were not prescribed an antithrombotic medication at discharge. The baseline characteristics of included and excluded AIS patients with active cancer are shown in Supplementary Table 1. The final study population comprised 135 patients, including 58 (43%) in the anticoagulant group and 77 (57%) in the antiplatelet group.

Per TOAST criteria, an undetermined cause (i.e., cryptogenic) was the leading mechanism in both treatment groups: 82% of patients in the anticoagulant group (*n* = 48/58) and 69% in the antiplatelet group (*n* = 53/77). In these cryptogenic cases, ESUS criteria were met in 100% of the anticoagulant group (*n* = 48/48) and 72% of the antiplatelet group (*n* = 38/53). Large-artery atherosclerosis was identified in 9% of patients treated with anticoagulation (*n* = 5/58) and 25% of those treated with antiplatelet therapy (*n* = 19/77). Other determined causes were present in 9% of the anticoagulant group (*n* = 5/58) and 2% of the antiplatelet group (*n* = 2/77). Small-vessel disease was only observed in the antiplatelet group (4%, *n* = 3/77).

Of the 58 patients treated with anticoagulation, 41 (71%) received LMWH, 4 (7%) received vitamin K antagonists, 8 (14%) received DOACs (edoxaban, rivaroxaban, or apixaban), and 5 received (9%) a combination of antiplatelet plus anticoagulant therapy. Among the 41 patients discharged with LMWH, 21 (51%) had died at 3-months, 14 (34%) were lost to follow-up, 4 (10%) were switched to DOAC, and 2 were still receiving LMWH (5%). Median follow-up time for long-term mortality was 495 days (IQR 57–1,029) for the overall cohort, 133 days (IQR 43–506) for the anticoagulant group, and 797 days (IQR 218–1,483) for the antiplatelet group.

**Figure 2:**
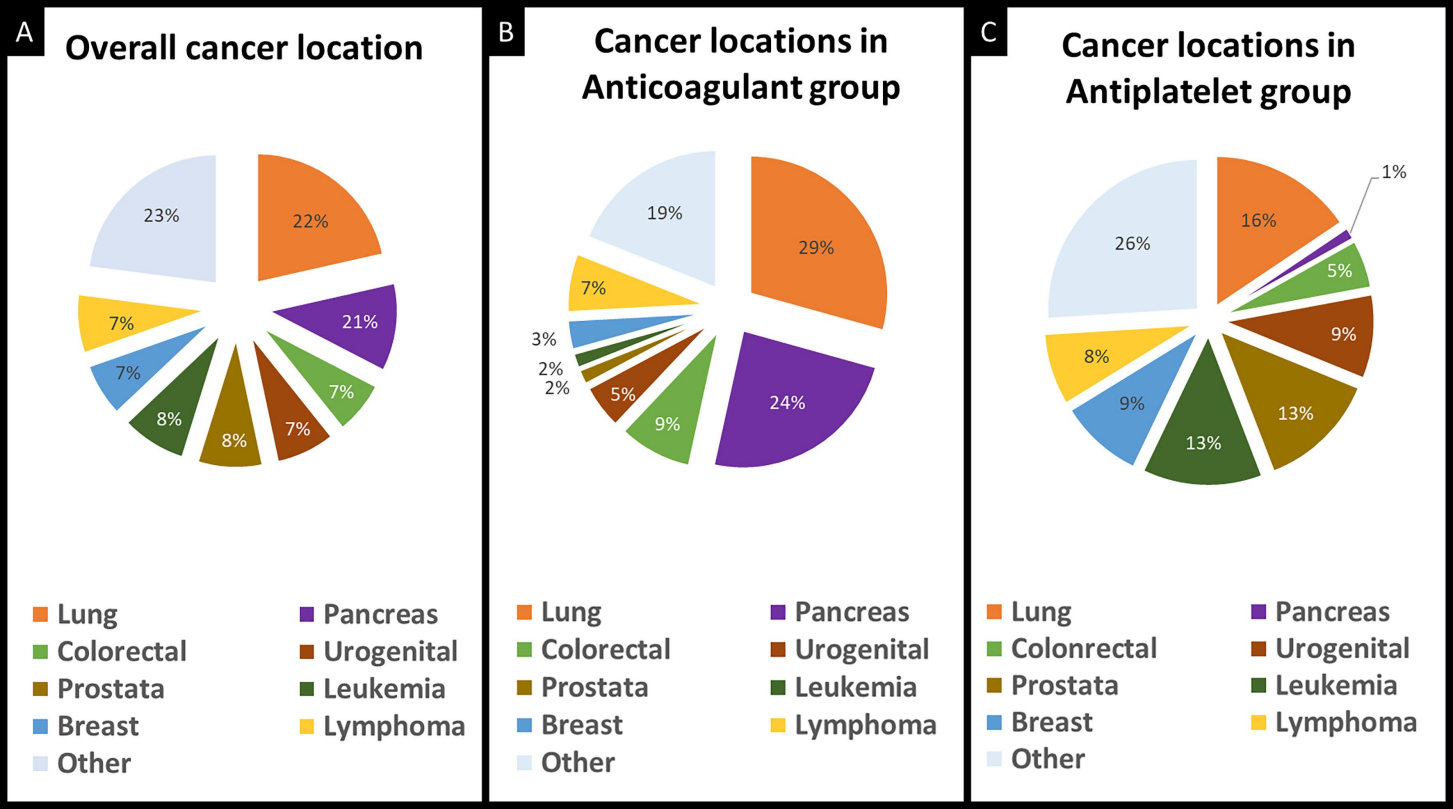
Distribution of primary cancer location in included patients. Primary cancer location in the overall study population **(A)**, in the anticoagulant group **(B)**, and in the antiplatelet group **(C)**.

Patients treated with anticoagulant therapy, compared to patients treated with antiplatelet therapy, were younger (69 years [IQR 62–75] versus 75 years [IQR 65–82], *p* = 0.01), had more multi-territory brain infarcts (47% versus 17%, *p* < 0.001), and more often had an ESUS mechanism (82% versus 50%, *p* < 0.001; Table 1). Data on the primary cancer site in the overall study population and stratified by the individual treatment groups are provided in Figure 2. The distribution of primary cancer sites differed between groups with higher rates of lung and pancreatic cancers in the anticoagulant group (*p* < 0.001). Additionally, patients in the anticoagulant group had a higher median cancer stage (4 [IQR 3–4] versus 3 [IQR 2–4], *p* < 0.001) and more frequent metastases at the time of AIS (72% versus 41%, *p* < 0.001). There were 23 patients with documented VTEs, of whom 22 were prescribed anticoagulant therapy at hospital discharge.

**Table 1:** Baseline characteristic data in included patients with active cancer and ischemic stroke stratified by antiplatelet versus anticoagulant therapy.

	All patients (*N* = 135)	Antiplatelet therapy (*N* = 77)	Anticoagulant therapy (*N* = 58)	*p*-value
Demographics				
Sex, female	60/135 (44)	31/77 (40)	29/58 (50)	0.30
Age at admission, median	72 (64–80)	75 (65–82)	69 (62–75)	0.01
Medical history				
Previous ischemic stroke	20/96 (21)	8/53 (15)	12/43 (28)	0.14
Hypertension	65/96 (68)	38/53 (72)	27/43 (63)	0.39
Diabetes Mellitus	21/95 (22)	10/52 (19)	11/43 (26)	0.47
Hyperlipidemia	66/95 (70)	38/52 (73)	28/43 (65)	0.50
Smoking history	28/90 (31)	15/49 (31)	13/41 (32)	1.00
Venous thromboembolic events				
Any venous thromboembolism	23/135 (17)	1/77 (1)	22/58 (38)	<0.001
Deep venous thrombosis	14/135 (10)	1/77 (1)	13/58 (22)	<0.001
Pulmonary embolism	16/135 (12)	0/77 (0.0)	16/58 (28)	<0.001
Stroke characteristics				
Independence before stroke (mRS ≤ 2)	54/135 (44)	30/33 (91)	24/27 (89)	1.00
Initial NIHSS, median	5 (2–9)	4 (2–8)	6 (3–10)	0.31
MRI during admission	59/80 (73)	36/48 (75)	23/32 (72)	0.80
Multi-territory infarct	40/135 (30)	13/77 (17)	27/58 (47)	<0.001
Stroke etiology according to TOAST				
Large-artery atherosclerosis	24/135 (18)	19/77 (25)	5/58 (9)	0.011
Cardioembolic	0/135 (0)	0/77 (0)	0/58 (0)	
Small-vessel disease	3/135 (2)	3/77 (4)	0/58 (0)	
Other determined cause	7/135 (5)	2/77 (2)	5/58 (9)	
Undetermined cause	101/135 (75)	53/77 (69)	48/58 (82)	
ESUS	86/135 (64)	38/77 (50)	48/58 (82)	<0.001
Cancer characteristics at time of AIS				
Cancer stage, median	4 (2–4)	3 (2–4)	4 (3–4)	<0.001
Distant metastases	65/135 (48)	27/77 (41)	38/58 (72)	<0.001
Baseline laboratory findings, median				
D-dimer in μg/L	2037 (684–9,118)	1,010 (495–2090)	8,536 (2080–13,726)	<0.001
Elevated D-Dimer (≥2037 μg/L)	48/74 (65)	12/32 (38)	36/42 (86)	<0.001
CRP in mg/L	3 (1–8)	4 (2–24)	18 (5–50)	0.005
Fibrinogen in g/L	3.1 (2.6–3.7)	3.3 (2.6–3.9)	2.7 (2.0–3.5)	0.003
LDH in U/l	396 (339–480)	387 (344–499)	650 (446–833)	<0.001
Platelet count in G/L	225 (186–271)	229 (191–267)	181 (144–250)	0.01
Hemoglobin in g/dL	13.8 (12.6–14.9)	13 (10.5–14.2)	11.8 (10.8–13.4)	0.25

Categorical variables are listed as number/total number (percentage) and continuous or ordinal variables are listed as median (interquartile range). CRP, C-reactive protein; ESUS indicates embolic stroke of undetermined source; LDH, Lactate dehydrogenase; mRS, modified Rankin Scale; NIHSS, National Institutes of Health Stroke Scale; VTE, venous thromboembolism.

Four patients in the antiplatelet group received intravenous thrombolysis before D-dimer sampling and were excluded from analyses including laboratory parameters. Compared to patients in the antiplatelet group, patients in the anticoagulant group had higher D-dimer levels in μg/L (median [IQR]: 8536 [2080–13,726] versus 1,010 [495–2,090], *p* < 0.001), higher CRP levels in mg/L (median [IQR]:18 [5–50] versus 4 [2–24], *p* = 0.01) and lower hemoglobin levels in g/dL (median [IQR]:11.8 [10.8–13.4] versus 13.0 [10.5–14.2], *p* = 0.03).

### Primary outcome

**Figure 3:**
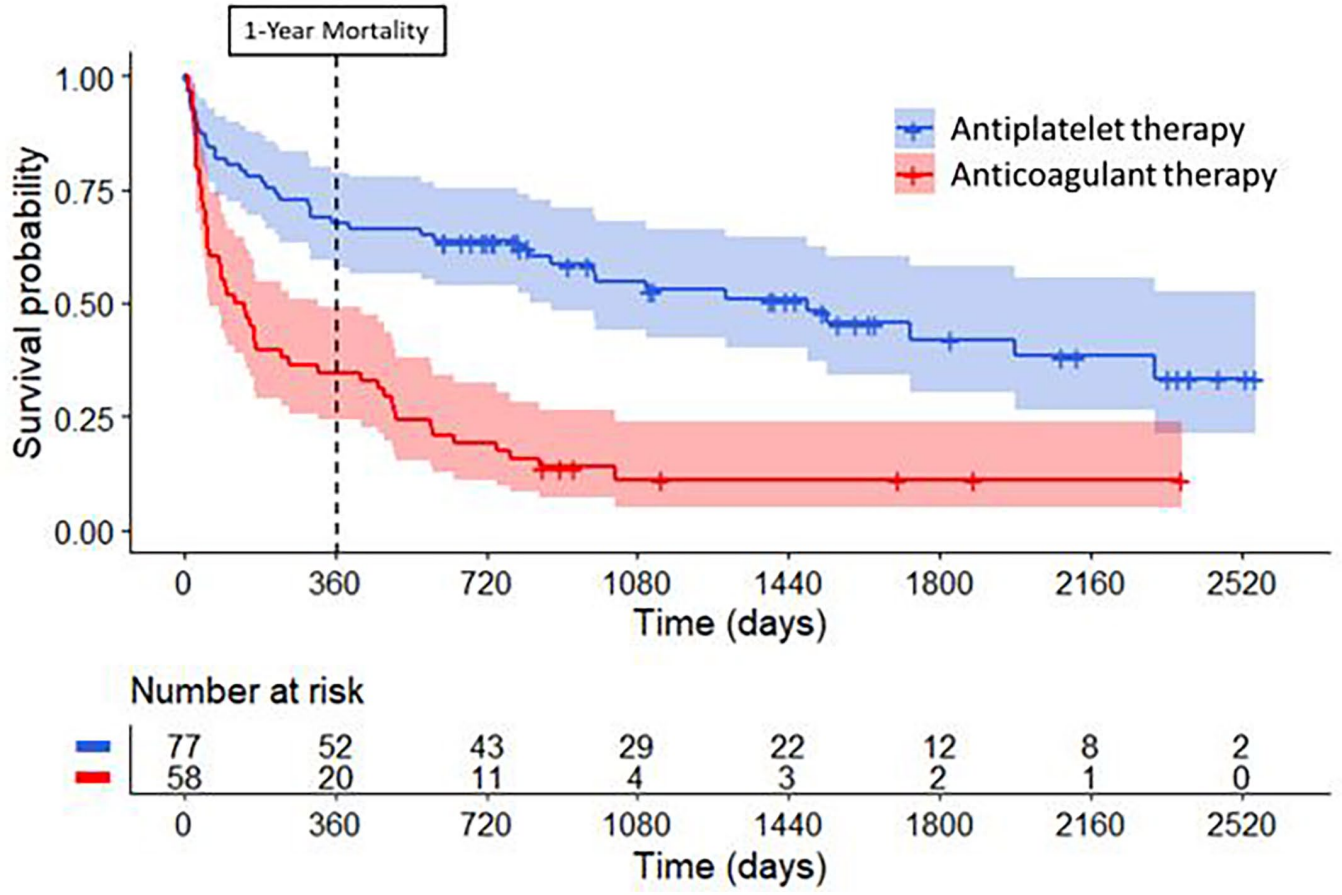
Long-term survival curves for patients with cancer treated with antiplatelet therapy or anticoagulant therapy for secondary stroke prevention. Compared to patients treated with antiplatelet therapy (in blue), patients treated with anticoagulant therapy (in red) had higher mortality rates at one year (log-rank test, *p* < 0.001) and during long-term follow-up (log-rank test, *p* < 0.001) after their index ischemic stroke.

**Figure 4:**
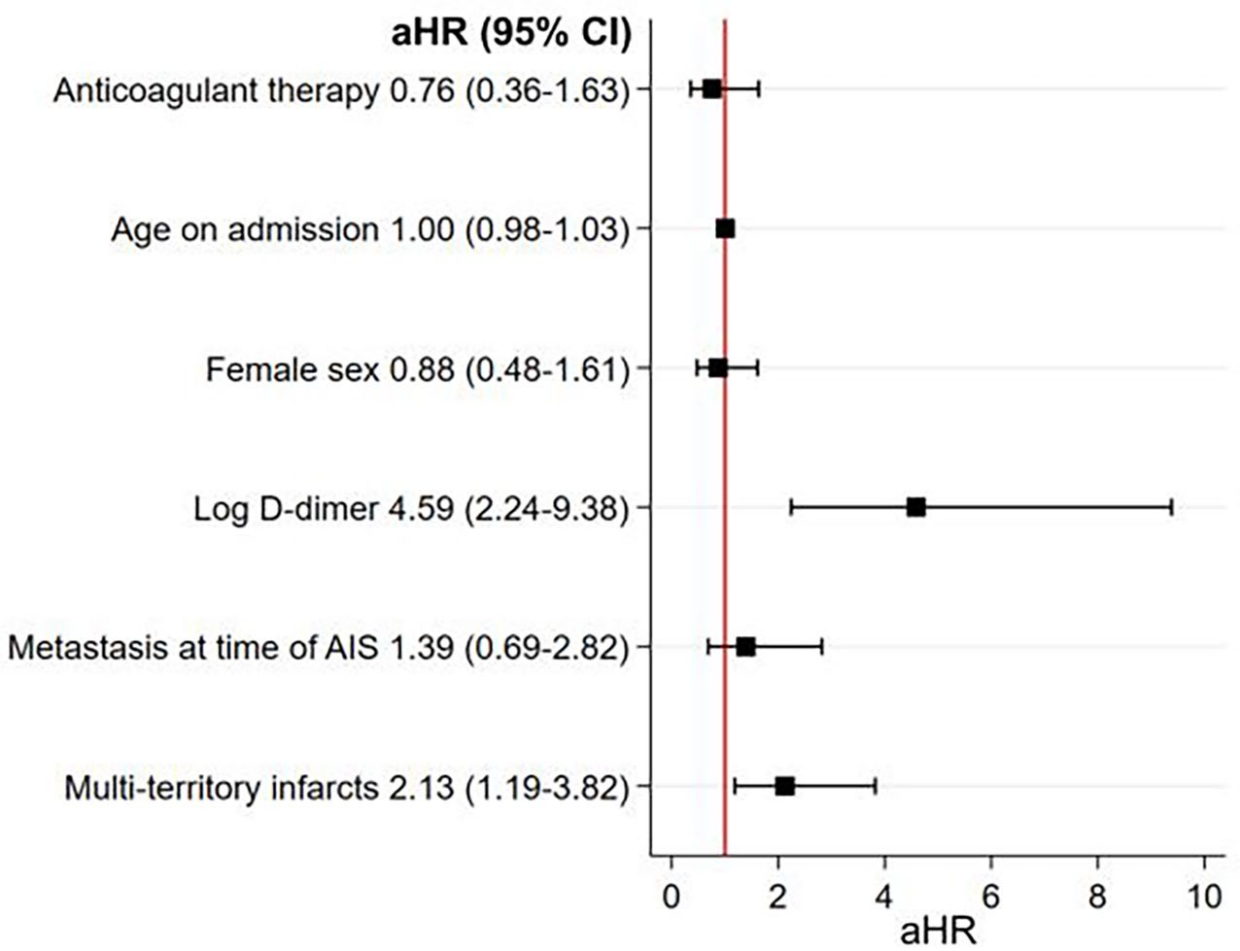
Multivariable model studying the association between antithrombotic treatment strategies at hospital discharge for AIS and 1-year mortality. There was no association between anticoagulant therapy, as compared to antiplatelet therapy, and 1-year mortality in the main analysis. Higher D-dimer levels and multi-territory brain infarcts were both strongly associated with 1-year mortality in these patients. D-dimer was abnormally distributed so it was log transformed. aHR, adjusted hazard ratio; AIS, acute ischemic stroke.

As depicted in Figure 3, the estimated cumulative 1-year mortality rate was higher in the anticoagulant group (66, 95% CI 53–77%) than in the antiplatelet group (33, 95% CI 23–44%) (log-rank test *p* < 0.001). In multivariable Cox regression analysis, anticoagulant use was associated with a similar 1-year mortality rate as antiplatelet use (aHR 0.76; 95% CI 0.36–1.63; *p* = 0.47) (Figure 4). Factors independently associated with 1-year mortality after AIS were initial D-dimer levels (aHR 4.59; 95% CI 2.24–9.38; *p* < 0.001) and multi-territory brain infarction (aHR 2.13; 95% CI 1.19–3.82; *p* = 0.01). After excluding five patients who received combined antiplatelet and anticoagulant therapy, no difference remained between groups for 1-year mortality (aHR 0.81; 95% CI 0.38–1.74; *p* = 0.59) (Supplementary Figure 1).

### Secondary outcomes

As shown in Table 2, patients in the anticoagulant group had a lower rate of good functional outcomes (mRS ≤ 2) at 90 days compared to those in the antiplatelet group (29% versus 66%, *p* = 0.003). After adjustment for potential confounders, there was no difference in 90-day good functional outcomes between study groups (aHR 1.36; 95% CI 0.58–3.19, *p* = 0.49, Supplementary Figure 2).

**Table 2:** Clinical outcomes in included patients with active cancer and AIS stratified by antiplatelet versus anticoagulant therapy.

	All patients (*N* = 135)	Antiplatelet therapy (*N* = 77)	Anticoagulant therapy (*N* = 58)	*p*-value
90-day follow-up				
Good functional outcomes (mRS ≤ 2)	39/78 (50)	29/44 (66)	10/34 (29)	0.003
Mortality rate	19/78 (24)	5/44 (11)	14/34 (41)	0.003
Recurrent AIS	3/66 (5)	3/43 (7)	0/23 (0)	0.55
Occurrence of sICH	0/66 (0)	0/43 (0)	0/23 (0)	NA
Major clinical bleeding besides ICH	0/135 (0)	0/43 (0)	0/23 (0)	NA
Clinically relevant non-major bleeding	1/135 (0.7)	1/43 (2.33)	0/23 (0)	1.00
Long-term follow-up				
Follow-up time for long-term mortality in days, median	495 (57–1,029)	797 (218–1,483)	133 (43–506)	<0.001
Mortality rate at one year	63/135 (47)	25/77 (33)	38/58 (66)	<0.001
Mortality rate in the long-term	121/135 (90)	40/77 (52)	51/58 (88)	<0.001
Follow-up time for cerebrovascular events in days, median	165 (80–707)	178 (99–940)	107 (40–232)	0.002
Recurrent AIS	11/135 (8)	6/77 (8)	5/58 (9)	1.00
Occurrence of sICH	0/135 (0)	0/77 (0)	0/58 (0)	NA
Major clinical bleeding besides ICH	1/135 (0.7)	0/77 (0)	1/58 (1.7)	0.43
Clinically relevant non-major bleeding	2/135 (1.5)	1/77 (1.3)	1/58 (1.7)	1.00

Categorical variables are listed as number/total number (percentage) and continuous or ordinal variables are listed as median (interquartile range). AIS, acute ischemic stroke; mRS modified Rankin Scale; sICH, symptomatic intracranial hemorrhage; ICH, intracranial hemorrhage.

At 90-days, 0% of patients in the anticoagulant group (*n* = 0/23) were diagnosed with recurrent AIS compared to 7% in the antiplatelet group (*n* = 3/43, *p* = 0.55). There were no sICHs in either group during the first 90 days of follow-up.

Patients treated with anticoagulant therapy had higher long-term mortality compared to patients treated with antiplatelet therapy (88, 95% CI 77–94% versus 52, 95% CI 41–63%, log-rank test *p* < 0.001). However, after adjustment for potential confounders, there was no difference in long-term mortality between the groups (aHR 1.29; 95% CI 0.67–2.47; *p* = 0.44) (Supplementary Figure 3).

The median total follow-up time for cerebrovascular events was 165 days (IQR 80–707) for the overall cohort, 107 days (IQR 40–232) for the anticoagulant group, and 178 days (IQR 99–940) for the antiplatelet group. After one year and also during the entire follow-up period, 9% (*n* = 5/58) of patients treated with anticoagulant therapy had a recurrent AIS compared to 8% (*n* = 6/77) of patients treated with antiplatelet therapy (aHR 0.49; 95% CI 0.08–2.83; *p* = 0.83, Supplementary Figure 4). There were no cases of sICH in either group during long-term follow-up. Major bleeding besides intracranial hemorrhage or clinically relevant non-major bleeding were reported in three patients (2% of the entire cohort), all occuring within one year after AIS, with no differences between treatment groups. When accounting for mortality as a competing risk, the recurrence of AIS did not differ between patients receiving anticoagulants and those receiving antiplatelet therapy (Supplementary results I).

### Subgroup analyses

Of the 135 index AIS, 86 (64%) were classified as ESUS. In the ESUS subgroup (Supplementary Table 2), patients treated with anticoagulant therapy were on average younger and more often had metastatic disease compared to those treated with antiplatelet therapy. After multivariable adjustment, anticoagulant therapy, compared to antiplatelet therapy, was associated with similar mortality at one year after AIS (aHR 0.51; 95% CI 0.21–1.22; *p* = 0.11, Supplementary Figure 5). In patients with ESUS and D-dimer levels above the median (2,037 μg/L) for the cohort (*n* = 35), there was no difference in 1-year mortality rates between antithrombotic treatment groups after multivariable adjustment (aHR 0.26; 95% CI 0.05–1.27; *p* = 0.10, Supplementary Figure 10). Results were similar in patients with ESUS and multiterritory infarcts (*n* = 29) (aHR 1.17; 95% CI 0.32–4.19; *p* = 0.81, Supplementary Figure 11).

### Sensitivity analyses

After exclusion from the initial study population of 22 patients with documented VTE treated with anticoagulant therapy at the time of index AIS hospital discharge, the risk of 1-year mortality remained similar between patients treated with anticoagulant therapy and those treated with antiplatelet therapy (aHR 0.65; 95% CI 0.28–1.47; *p* = 0.30, Supplementary Figure 6). Of the remaining 36 patients discharged on anticoagulant therapy, 24 (67%) were treated with LMWH, 3 (8%) with vitamin K antagonists, 7 (19%) with DOACs, and 2 (6%) with combination therapy. After excluding the same 22 patients with documented VTE treated with anticoagulant therapy at index AIS hospital discharge from the ESUS cohort, 1-year mortality remained similar between antithrombotic treatment groups (aHR 0.38; 95% CI 0.14–1.05; *p* = 0.06, Supplementary Figure 7).

### Inverse probabilty of treatment weighting analyses

In the second round of analyses employing IPTW, the stabilized weights exhibited a near-normal distribution centered around one, albeit with a few weights surpassing two, which could have a significant impact on study outcomes (Supplementary Figure 8).

**Figure 5:**
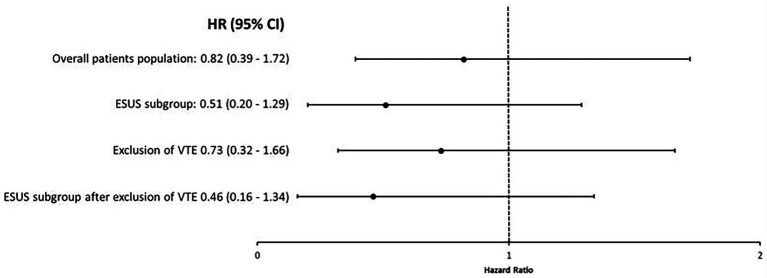
Univariable analyses studying the association between anticoagulant versus antiplatelet therapy at hospital discharge for AIS and 1-year mortality using IPTW with stabilized weights in the different study groups. There was no association between anticoagulant therapy, as compared to antiplatelet therapy, and 1-year mortality in weighted analysis using IPTW. This was the case in the overall study population as well as in patient subgroups. CI, confidence interval; ESUS, embolic stroke of undetermined source; HR, Hazard ratio; IPTW, inverse probability of treatment weighting and VTE, venous thromboembolism.

The IPTW analysis for 1-year mortality (Figure 5) corroborated the main analyses for the primary AIS cohort (HR 0.82; 95% CI 0.39–1.72, *p* = 0.61) and the ESUS subgroup (HR 0.51; 95% CI 0.20–1.29; *p* = 0.15). These results were unchanged after excluding patients with documented VTE from the primary cohort (HR 0.73; 95% CI 0.32–1.66; *p* = 0.46) and the ESUS subgroup (HR 0.46; 95% CI 0.16–1.34; *p* = 0.30).

## Discussion

Among 135 patients with active cancer and non-cardioembolic AIS at a comprehensive stroke center in Switzerland, short- and long-term clinical outcomes did not differ between patients treated with anticoagulant therapy at discharge and those treated with antiplatelet therapy. Study groups differed substantially, as treatment with anticoagulation was associated with more advanced and historically aggressive cancer types with predilections for hypercoagulability. These neutral findings persisted when analyses were limited to patients with ESUS and when excluding patients with VTE.

In the absence of specific guidelines and robust prospective data on secondary prevention in patients with AIS and active cancer, neurologists often rely on theoretical considerations and institutional practice patterns to guide treatment decisions (6). As prothrombotic processes play a central role in many cancer-related strokes, some neurologists favor empiric anticoagulant therapy in these patients (25). Some experts have argued that anticoagulant therapy may preferentially benefit cancer patients whose AIS is due to cerebral intravascular coagulation, non-bacterial thrombotic endocarditis, or paradoxical embolism (26). In patients with cancer-mediated hypercoagulability, it is purported that high thrombin levels promote the conversion of fibrinogen to fibrin and platelet activation, and this may be more effectively targeted by anticoagulant therapy than by antiplatelet therapy (26). D-dimer, a degradation product of cross-linked fibrin, is widely used as a surrogate marker of hypercoagulability in patients with cancer-related stroke (27). The OASIS-Cancer study demonstrated a reduction in 1-year mortality among patients whose D-dimer levels were effectively lowered with anticoagulant therapy (8). However, this study lacked an antiplatelet arm, and provided little information on cancer treatments administered, which, by targeting the underlying cancer driving hypercoagulability, may influence clinical outcomes more than the type of antithrombotic therapy selected (8). D-dimer levels have also been shown to correlate with microemboli on transcranial Doppler ultrasound, another marker of hypercoagulability (28, 29). In contrast, patients with active cancer and AIS also face an increased risk of major bleeding, approaching 20% at 1-year in prospective studies (30), and anticoagulant therapy is known to increase the risk of bleeding compared with antiplatelet therapy (31). These countervailing considerations may offset any potential reduction in thromboembolic risk with anticoagulation.

Given our neutral findings and the existing data (26), the potential efficacy of antiplatelet therapy in cancer-related stroke warrants further investigation. Large vessel occlusive thrombi endovascularly retrieved from patients with active cancer and AIS have been shown to be platelet-rich and erythrocyte-poor, particularly in subgroups with confirmed nonbacterial thrombotic endocarditis or ESUS (32). The MOST-Cancer study showed that P-selectin, a marker of platelet activation, is significantly elevated in patients with active cancer and AIS compared with cancer-only and stroke-only controls (29). This prospective study also found that P-selectin was predictive of major thromboembolic events or death in the cancer-related stroke group (30).

Few studies have compared clinical outcomes in cancer-related stroke by antithrombotic treatment strategy. In a retrospective analysis of 172 patients with active cancer and AIS at a comprehensive cancer center in New York, the rates of recurrent thromboembolism or death did not differ between patients treated with antiplatelet versus anticoagulant therapy (33). A subgroup analysis of the NAVIGATE ESUS trial evaluating 543 ESUS patients with any history of cancer reported no difference in the risk of recurrent AIS or mortality between patients treated with rivaroxaban versus aspirin (10). However, only 9% of patients in this post-hoc analysis had their cancer diagnosed in the year prior to the index AIS, so many of the included cancers were likely inactive during follow-up. A *post hoc* analysis investigated 137 patients with history of cancer in the ARCADIA trial, which compared apixaban to aspirin in patients with cryptogenic stroke and biomarker evidence for atrial cardiopathy (9). This study showed no significant difference in the risk of major ischemic and hemorrhagic events between antithrombotic treatment groups. However, once again, cancer status at the time of stroke was unknown, making it difficult to draw conclusions about optimal secondary stroke prevention for patients with active cancer based on these data. The TEACH trial randomized 20 patients with cancer-related stroke to subcutaneous enoxaparin (1 mg/kg twice daily) or oral aspirin (81-325 mg daily), and found similar rates of adverse clinical outcomes between groups (21). However, TEACH focused on demonstrating the feasibility of randomizing patients with cancer and AIS to these competing antithrombotic strategies, which it did, but was not powered for safety or efficacy.

In the current study, because of our institution’s guidelines, LMWH was the preferred anticoagulant for patients with ischemic stroke attributed to paraneoplastic coagulopathy. The continued use of LMWH beyond the acute phase appears to be inappropriate, as only 7% of patients with available follow-up (*n* = 2/27) remained on LMWH at 3 months in our study. This lack of compliance for LMWH is further supported by the TEACH trial, in which 40% of patients initially assigned to LMWH switched to aspirin due to discomfort with injections (21). The results of the current analysis support the existence of clinical equipoise between anticoagulant and antiplatelet therapy for the treatment of cancer-related stroke, and further support calls for randomized trials to determine the optimal strategy. An important consideration when deciding on the antithrombotic management of this patient group is the presumed stroke mechanism. As many ESUS in cancer patients are attributed to paraneoplastic coagulopathy, this subgroup may be the most likely to preferentially benefit from anticoagulant therapy. In this vein, when we analyzed ESUS patients only, there was a trend towards lower 1-year mortality with anticoagulation (aHR 0.51; 95% CI 0.2–1.2). Furthermore, this nonsignificant trend was even stronger in ESUS patients with elevated D-dimer levels (aHR 0.26; 95% CI 0.05–1.27).

Our study has several limitations. Firstly, confounding by indication bias is possible because patients treated with anticoagulant therapy were more likely to have elevated D-dimer levels, multi-territory brain infarcts, and metastatic disease at the time of AIS, all of which are associated with cancer-associated hypercoagulability and worse outcomes after cancer-related stroke (30). The presence of these markers of high tumor burden and paraneoplastic coagulopathy may have driven clinicians to preferentially prescribe anticoagulant therapy, thus potentially biasing our results. In fact, patients treated with anticoagulant therapy had a higher average cancer stage and frequency of metastases than patients treated with antiplatelet therapy, and these factors likely led to higher mortality and shorter follow-up time in the anticoagulant group through confounding, even despite multivariable adjustments. In such scenarios, IPTW is the recommend method for addressing casual inference, but residual confounding remains a concern (24, 34). Secondly, our study was conducted before widespread DOAC use in patients with cancer at a single comprehensive stroke center in Switzerland with predominantly Caucasian patients, which limits generalizability. Future studies should investigate more contemporary and heterogeneous patient cohorts. Thirdly, because of the retrospective study design, we relied on electronic health records to determine clinical data, which may have led to measurement error in patients’ clinical characteristics as well as missed clinical outcomes during follow-up. In particular, rates of recurrent AIS and bleeding outcomes may have been underestimated because incident diagnoses made in the outpatient setting or at other medical centers may have been missed. Fourthly, our study exposure was the type of antithrombotic therapy administered at hospital discharge, a single timepoint, and antithrombotic prescription patterns may have changed during follow-up due to clinical events and patient and physician preferences. For instance, some patients initially treated with antiplatelet therapy at hospital discharge may have been subsequently treated with anticoagulant therapy during follow-up and vice versa. Fifthly, our cohort was a convenience sample and the CIs for some analyses were wide and possibly underpowered to detect clinically meaningful differences between treatment groups. This includes recurrent AIS and long-term mortality. Sixthly, our database lacked data on the specific cause of death. Seventhly, we did not perform a formal power calculation or adjust for multiple comparisons meaning that all results should be considered hypothesis-generating.

## Conclusion

In our study, there were no differences in functional outcomes, mortality, or recurrent AIS in patients with active cancer and AIS treated with anticoagulant therapy versus those treated with antiplatelet therapy. These data combined with those from existing studies as well as the lack of clear recommendations by major guidelines highlight the need for dedicated fully-powered clinical trials to determine the optimal antithrombotic strategy for the secondary prevention of cancer-related stroke.

## Data Availability

The data analyzed in this study is subject to the following licenses/restrictions: the dataset used in this study is derived from the Swiss Stroke Register (SSR). Access to the data requires a formal application and approval from the relevant authorities. The SSR dataset is of high quality, ensuring robust and reliable statistical analyses. Furthermore, the dataset is well-documented, allowing for full reproducibility of the statistical analyses performed in this study. Information about the dataset can be found on https://www.neurovasc.ch/fileadmin/files/arbeitsgruppen/SSR_Data_management_04.07.2024.pdf. Requests to access these datasets should be directed to https://kontaktformular.dkfbasel.ch/.
