# Supplementary material for: Anticoagulant versus antiplatelet treatment for secondary stroke prevention in patients with active cancer
Source: Front Neurol. 2025 Sep 16;16:1530775. doi: 10.3389/fneur.2025.1530775 (PMC12479245; doi:10.3389/fneur.2025.1530775)
Supplement: Supplementary file 1 [file Supplementary_file_1.docx]

**SUPPLEMENTARY MATERIAL**

**Anticoagulant versus Antiplatelet Treatment for Secondary Stroke Prevention in Patients with Active Cancer**

**Supplemental methods I**

Major bleeding other than intracranial hemorrhage was defined per modified ISTH criteria as any non-fatal, non-intracranial bleeding event that resulted in a drop in hemoglobin of ≥2 g/dL, required transfusion of ≥2 units of blood, or involved a critical site such as intraspinal, intraocular (with vision loss), pericardial, intra-articular, intramuscular with compartment syndrome, or retroperitoneal locations^1^. Clinically relevant non-major bleeding (CRNMB) was defined per ISTH criteria as overt bleeding that did not meet criteria for major bleeding but required medical intervention, led to unscheduled contact with a healthcare provider, resulted in interruption of antithrombotic therapy, or caused discomfort or impairment of daily activities^2^.

**Supplemental methods II**

To assess the specific hazards for recurrent acute ischemic stroke (AIS) or the occurrence of symptomatic intracranial hemorrhage (sICH) according to the antithrombotic therapy prescribed at discharge, we performed competing-risks regression analysis using overall mortality as the competing-risk event. Subhazard ratios (SHR) and their associated 95% confidence interval (CI) were reported. Univariate and multivariate analyses, including the same co-variables as in the main analyses, were conducted. Since the follow-up data for cerebrovascular events and mortality came from two different sources (the local electronic health record and the Swiss Population Registry, respectively), only patients with occurrence of cerebrovascular events or with a time difference < 20% between the last documented follow-ups were considered for these analyses.

**Supplemental results I**

Of the 135 patients included in the study, 60 were excluded from the competing-risk analysis due to a time difference between both follow-up ≥ 20%. In the remaining patients, 19 patients (25%) stayed alive without any outcome events, 11 (15%) experienced a recurrent AIS, none (0%) experienced a sICH, and 45 (60%) died without having any new cerebrovascular event.
When accounting for mortality as a competing risk, the recurrence of AIS did not differ between patients receiving anticoagulants and those receiving antiplatelet therapy. In the univariate analysis, the SHR was 0.88 (95% CI 0.27-2.86; P=0.83), and in the multivariate analysis, the adjusted SHR was 0.37 (95% CI 0.10-1.30; P=0.12, eFigure XI).

**References Supplementary File:**

1. Schulman, S., & Kearon, C. (2005). Definition of major bleeding in clinical investigations of antihemostatic medicinal products in non-surgical patients. In *Journal of Thrombosis and Haemostasis* (Vol. 3, Issue 4). <https://doi.org/10.1111/j.1538-7836.2005.01204.x>
2. Kaatz, S., Ahmad, D., Spyropoulos, A. C., & Schulman, S. (2015). Definition of clinically relevant non-major bleeding in studies of anticoagulants in atrial fibrillation and venous thromboembolic disease in non-surgical patients: Communication from the SSC of the ISTH. *Journal of Thrombosis and Haemostasis*, *13*(11). https://doi.org/10.1111/jth.13140

**eTable I** - Baseline characteristics and outcomes in patients with acute ischemic stroke and active cancer included and excluded from analyses**.**

|  | All patients  (N= 306) | Included patients (n=135) | Excluded patients (n=171) | p-value |
| --- | --- | --- | --- | --- |
| **Demographics** | | | | |
| Sex, female | 118/306 (39) | 60/135 (44) | 58/171 (34) | 0.08 |
| Age at admission, median | 74 (67-82) | 72 (64-80) | 76 (69-83) | 0.03 |
| **Medical history** | | | | |
| Previous ischemic stroke | 45/242 (19) | 20/96 (21) | 25/146 (17) | 0.50 |
| Atrial fibrillation | 78/242 (32) | 0/96 (0) | 78/146 (53) | <0.001 |
| Dichotomized type of antithrombotic drugs at discharge | | | | |
| Anticoagulant | 136/270 (50) | 58/135 (43) | 78/135 (58) | 0.02 |
| Antiplatelet treatment | 134/270 (50) | 77/135 (57) | 57/135 (42) |  |
| Detailed type of antithrombotic drugs at discharge | | | | |
| No treatment | 36/306 (12) | 0/135 (0) | 36/171 (21) | <0.001 |
| Aspirin | 82/306 (27) | 51/135 (38) | 31/171 (18) |  |
| Clopidogrel | 26/306 (8) | 12/135 (9) | 14/171 (8) |  |
| Dual antiplatelet therapy | 26/306 (8) | 14/135 (10) | 12/171 (7) |  |
| Vitamin K antagonists | 7/306 (2) | 4/135 (3) | 3/171 (2) |  |
| Edoxaban | 13/306 (4) | 4/135 (3) | 9/171 (5) |  |
| Rivaroxaban | 10/306 (3) | 2/135 (1) | 8/171 (5) |  |
| Dabigatran | 6/306 (2) | 0/135 (0) | 6/171 (4) |  |
| Apixaban | 21/306 (7) | 2/135 (1) | 19/171 (11) |  |
| Low molecular weight heparin | 65/306 (21) | 41/135 (31) | 24/171 (14) |  |
| Combined anticoagulant and antiplatelet therapy | 14/306 (6) | 5/135 (4) | 9/171 (5) |  |
| **Stroke characteristics** |  |  |  |  |
| Independence before stroke (mRS ≤ 2) | 126/147 (86) | 54/60 (90) | 72/87 (83) | 0.24 |
| Initial NIHSS, median | 6 (2-13) | 5 (2-9) | 7 (2-15) | 0.03 |
| Stroke etiology according to TOAST | | | | |
| Large-artery atherosclerosis | 39/306 (13) | 24/135 (18) | 15/171 (9) | <0.001 |
| Cardioembolic | 87/306 (29) | 0/135 (0) | 87/171 (51) |  |
| Small-vessel disease | 3/306 (1) | 3/135 (2) | 0/171 (0) |  |
| Other determined cause | 13/306 (4) | 7/135 (5) | 6/171 (4) |  |
| Undetermined etiology | 163/306 (53) | 101/135 (75) | 62/171 (36) |  |
| ESUS, No./total No. (%) | 142/306 (46) | 86/135 (64) | 56/171 (33) | <0.001 |
| **Cancer characteristics at time of AIS** | | | | |
| Cancer stage, median | 4 (2-4) | 4 (2-4) | 3 (2-4) | 0.005 |
| Distant metastases | 110/275 (40) | 71/135 (53) | 39/140 (28) | <0.001 |
| D-dimer in µg/L | 1826 (687-6852) | 2037 (684-9118) | 1382 (664-4174) | 0.15 |
| **90-day follow-up** | | | | |
| Good functional outcomes (mRS ≤2) | 90/183 (49) | 39/78 (50) | 51/105 (49) | 0.88 |
| Mortality rate | 44/182 (24) | 19/78 (24) | 25/104 (24) | 1.0 |
| Recurrent AIS | 8/154 (52) | 3/66 (5) | 5/88 (6) | 1.0 |
| Occurrence of sICH | 0/154 | 0/66 (0) | 0/88 (0) | NA |
| **Long-term follow-up** | | | | |
| Mortality rate at one year | 148/273 (54) | 63/135 (47) | 85/138 (62) | 0.01 |
| Mortality rate in the long-term | 251/273 (92) | 121/135 (90) | 130/138 (94) | 0.19 |
| Categorical variables are listed as number/ total number (percentage) and continuous or ordinal variables are listed as median (interquartile range). Abbreviations: AIS, acute ischemic stroke; ESUS, embolic stroke of undetermined source; mRS, modified Rankin Scale; NIHSS, National Institutes of Health Stroke Scale; sICH, symptomatic intracerebral hemorrhage. | | | | |

**eTable II** - Baseline characteristics data in ESUS patients with active cancer stratified by antiplatelet versus anticoagulant therapy.

|  | All patients  (N= 86) | Antiplatelet therapy (N=38) | Anticoagulant therapy (N=48) | p-value |
| --- | --- | --- | --- | --- |
| **Demographics** | | | | |
| Sex, female | 46/86 (54) | 20/38 (53) | 26/48 (54) | 1.00 |
| Age at admission median | 71 (63-77) | 74 (65-79) | 67 (62-74) | 0.03 |
| **Medical history** | | | | |
| Previous ischemic stroke | 12/60 (20) | 2/24 (8) | 10/36 (28) | 0.10 |
| **Venous thromboembolic events** | | | | |
| Any venous thromboembolism | 30/86 (35) | 1/38 (3) | 29/48 (60) | <0.001 |
| Deep venous thrombosis | 14/86 (16) | 1/38 (3) | 13/48 (27) | 0.002 |
| Pulmonary embolism | 16/86 (19) | 0/38 (0) | 16/48 (33) | <0.001 |
| **Stroke characteristics** |  |  |  |  |
| Independence before stroke (mRS ≤ 2) | 33/38 (87) | 13/16 (81) | 20/22 (91) | 0.63 |
| Initial NIHSS, median | 6 (3-10) | 6 (3-10) | 6 (3-10) | 1.00 |
| MRI during admission | 38/50 (76) | 19/23 (82) | 19/27 (70) | 0.34 |
| Multi-territory infarct | 36/86 (42) | 12/38 (83) | 24/48 (50) | 0.12 |
| **Cancer characteristics at time of AIS** | | | | |
| Cancer stage, median | 3 (4-4) | 3 (2-4) | 4 (4-4) | <0.001 |
| Distant metastases | 51/86 (59) | 15/38 (39) | 36/48 (75) | <0.001 |
| **90-day follow-up** | | | | |
| Good functional outcomes (mRS ≤2) | 20/50 (40) | 14/23 (61) | 6/27 (22) | 0.009 |
| Mortality rate | 16/50 (32) | 3/23 (13) | 13/27 (48) | 0.01 |
| Recurrent AIS | 2/39 (5) | 2/22 (9) | 0/17 (0) | 0.50 |
| Occurrence of sICH | 0/39 (0) | 0/22 (0) | 0/17 (0) | NA |
| **Long-term follow-up** | | | | |
| Mortality rate at one year | 38/86 (44) | 16/38 (50) | 32/48 (67) | 0.03 |
| Mortality rate in the long-term | 79/86 (92) | 33/38 (87) | 46/48 (96) | 0.23 |
| Recurrent AIS in the long-term | 8/86 (9) | 4/38 (11) | 4/48 (8) | 0.73 |
| Occurrence of sICH in long-term | 0/86 (0) | 0/38 (0) | 0/48 (0) | NA |
| Categorical variables are listed as number/total number (percentage) and continuous or ordinal variables are listed as median (interquartile range).  Abbreviations: AIS, acute ischemic stroke; mRS modified Rankin Scale; MRI magnetic resonance imaging; NIHSS, National Institutes of Health Stroke Scale; sICH, symptomatic intracerebral hemorrhage. | | | | |


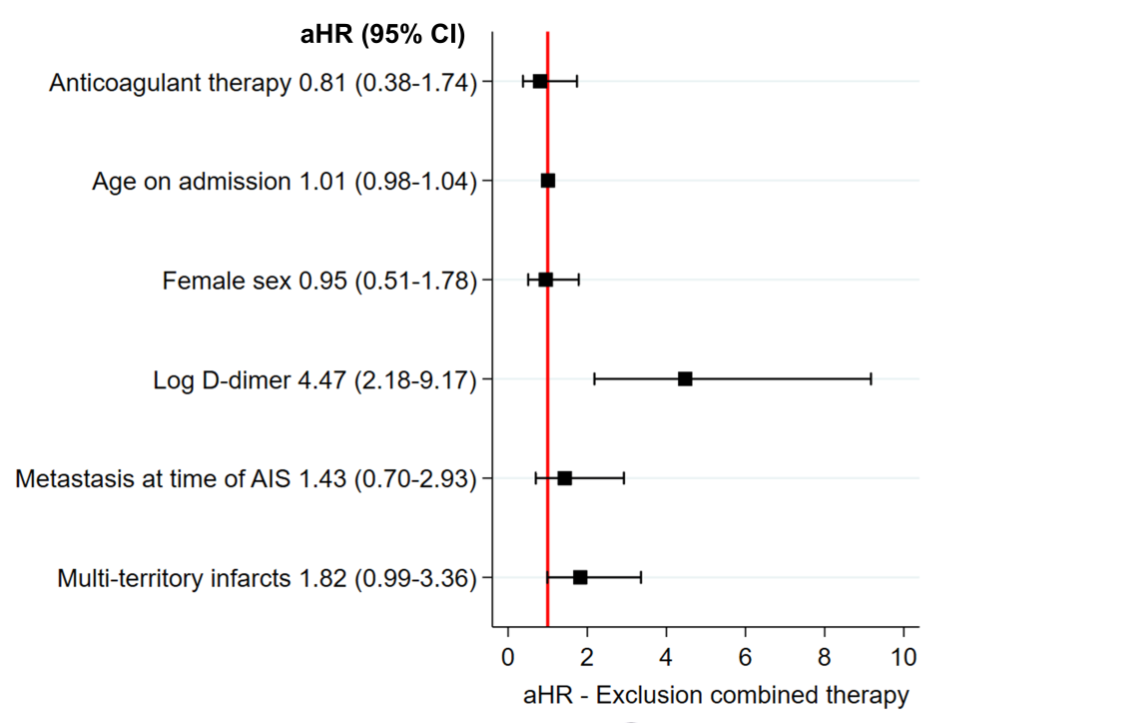


**eFigure I** – Multivariable model studying the association between antithrombotic treatment strategies at hospital discharge and mortality within one year after the exclusion of patients receiving combined anticoagulant and antiplatelet therapy.

There was no association between anticoagulant therapy, as compared to antiplatelet therapy, and one year mortality in this analysis. Higher D-dimer levels and multi-territory brain infarcts were both strongly associated with long-term mortality in these patients. D-dimer was abnormally distributed so it was log transformed. Abbreviations: aHR, adjusted hazard ratio; AIS, acute ischemic stroke.


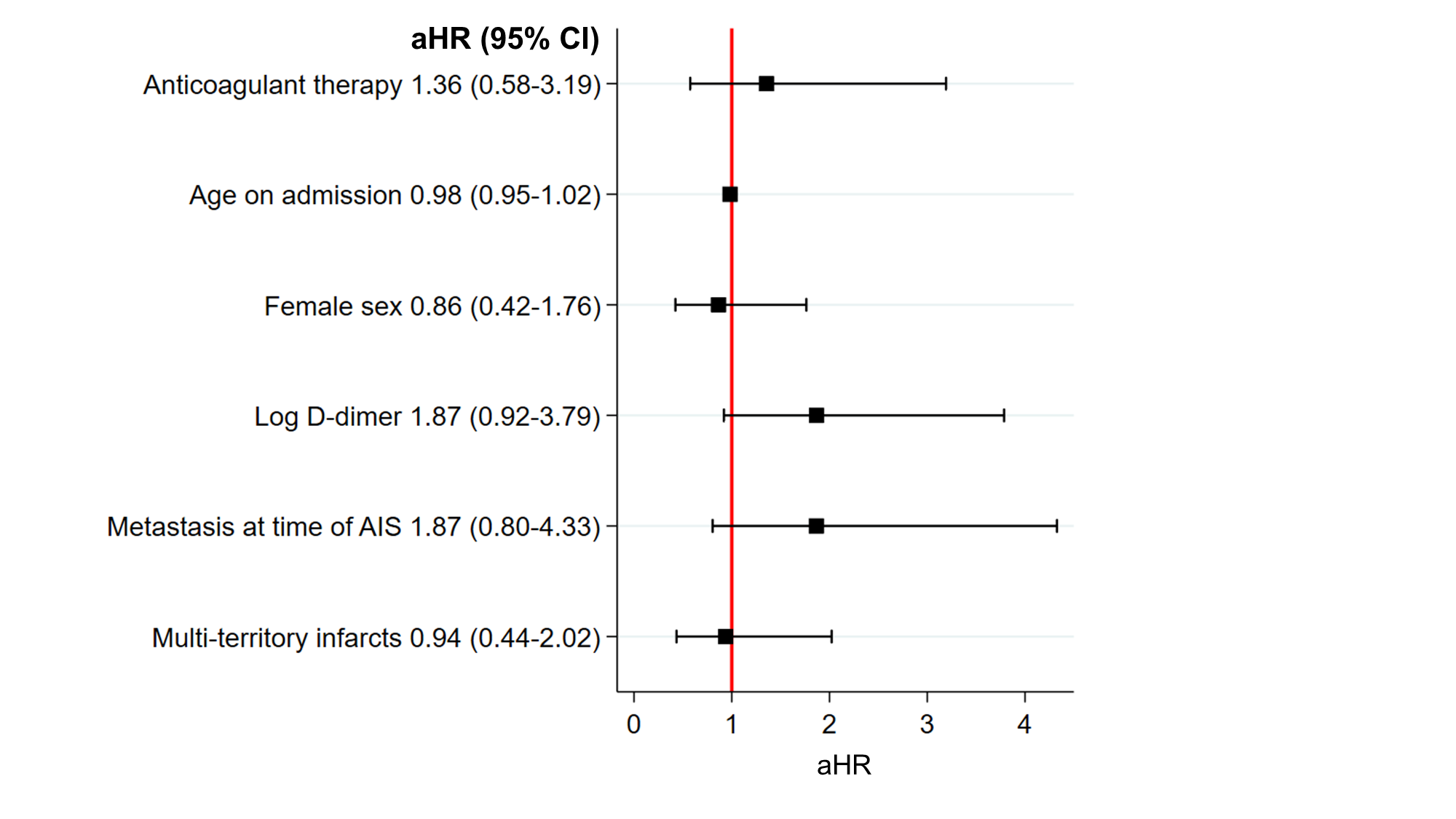


**eFigure II** – Multivariable model studying the association between antithrombotic treatment strategies at hospital discharge for AIS and 90-day good functional outcomes (mRS ≤2).

There was no association between anticoagulant therapy, as compared to antiplatelet therapy, and 90-day good functional outcomes in this analysis. D-dimer was abnormally distributed so it was log transformed. Abbreviations: aHR, adjusted hazard ratio; AIS, acute ischemic stroke; mRS, modified Rankin Scale.


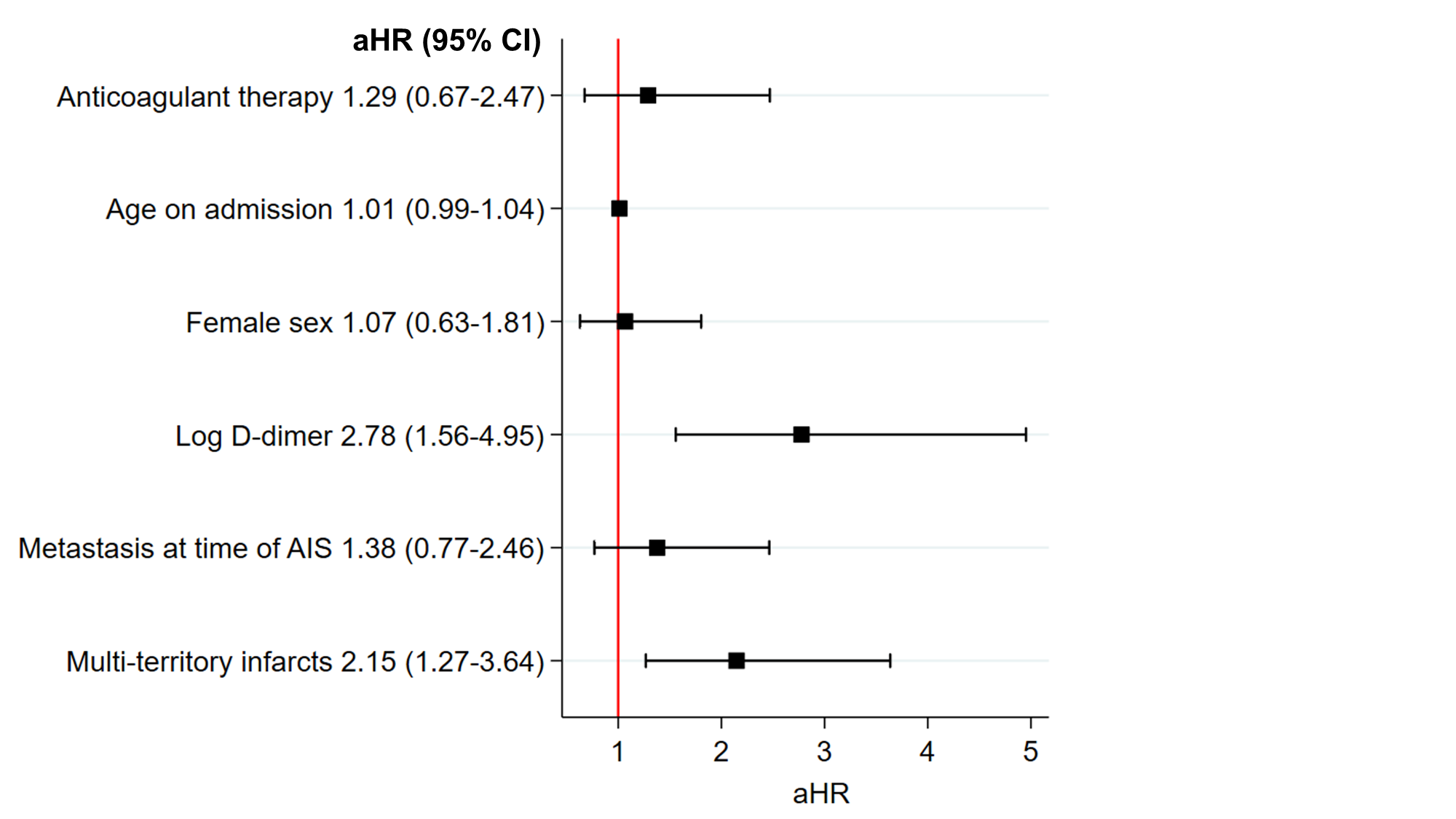


**eFigure III** – Multivariable model studying the association between antithrombotic treatment strategies at hospital discharge for AIS and long-term mortality.

There was no association between anticoagulant therapy, as compared to antiplatelet therapy, and long-term mortality in this analysis. Higher D-dimer levels and multi-territory brain infarcts were both strongly associated with long-term mortality in these patients.
D-dimer was abnormally distributed so it was log transformed. Abbreviations: aHR, adjusted hazard ratio; AIS, acute ischemic stroke.


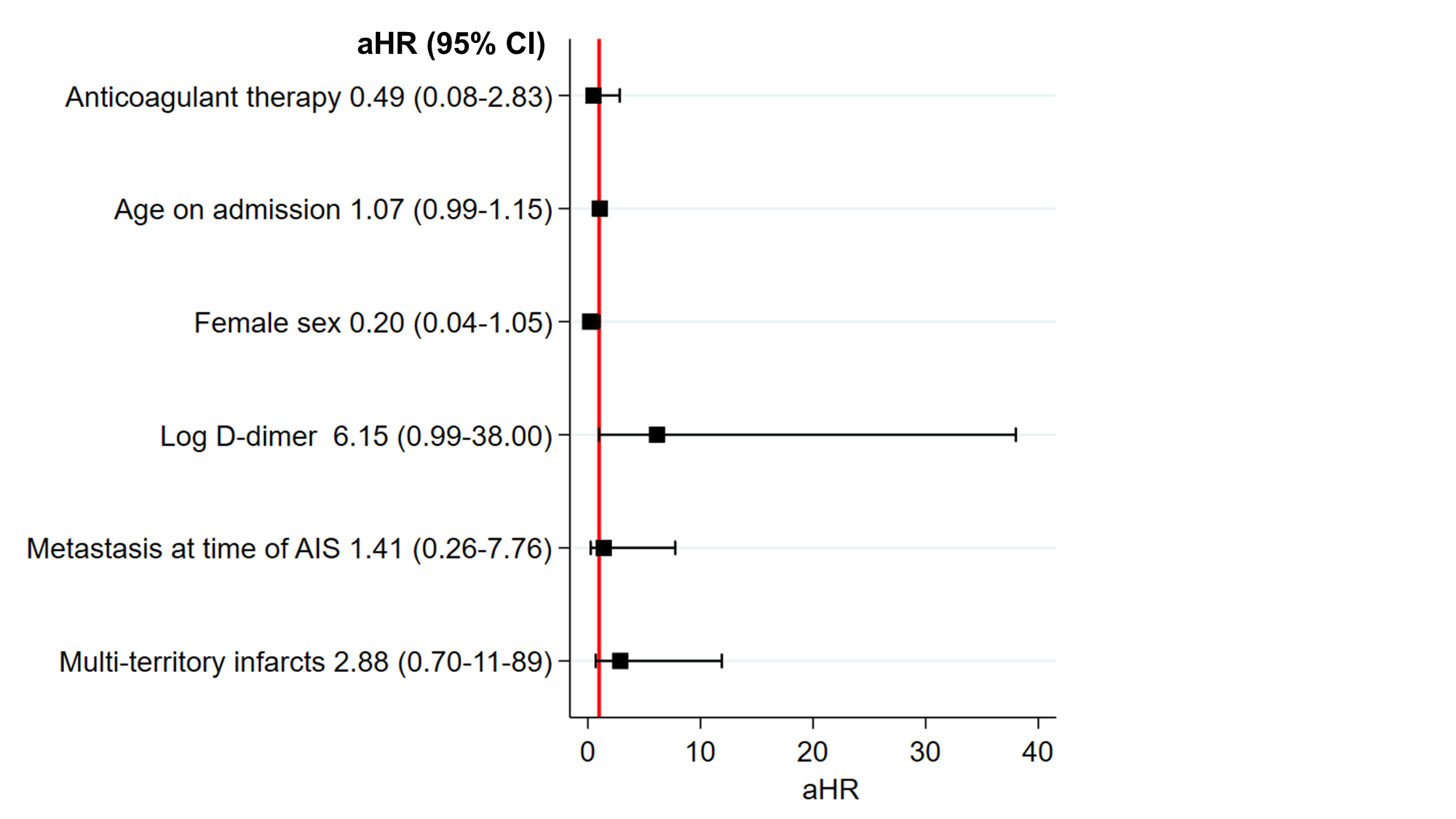


**eFigure IV** – Multivariable model studying the association between antithrombotic treatment strategies at hospital discharge for AIS and recurrent AIS during long-term follow-up.

There was no association between anticoagulant therapy, as compared to antiplatelet therapy, and recurrent AIS during long-term follow-up in this analysis. D-dimer was abnormally distributed so it was log transformed. Abbreviations: aHR, adjusted hazard ratio and AIS, acute ischemic stroke.


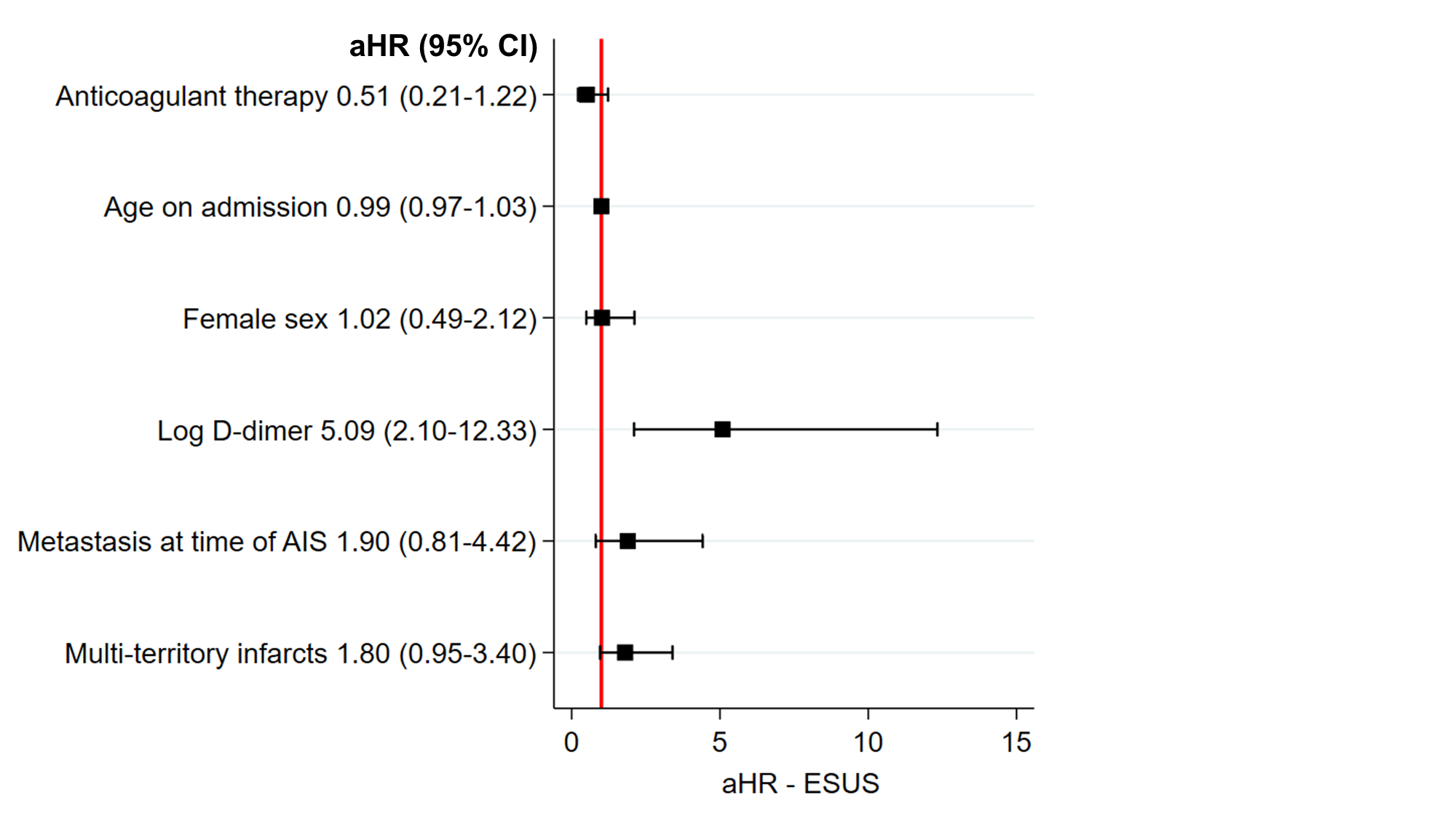


**eFigure V** – Multivariable model studying the association between antithrombotic treatment strategies at hospital discharge for AIS and 1-year mortality in the ESUS subgroup.

There was no association between anticoagulant therapy, as compared to antiplatelet therapy, and 1-year mortality in this analysis. D-dimer was abnormally distributed so it was log transformed. Abbreviations: aHR, adjusted hazard ratio; AIS, acute ischemic stroke and ESUS, embolic stroke of undetermined source.


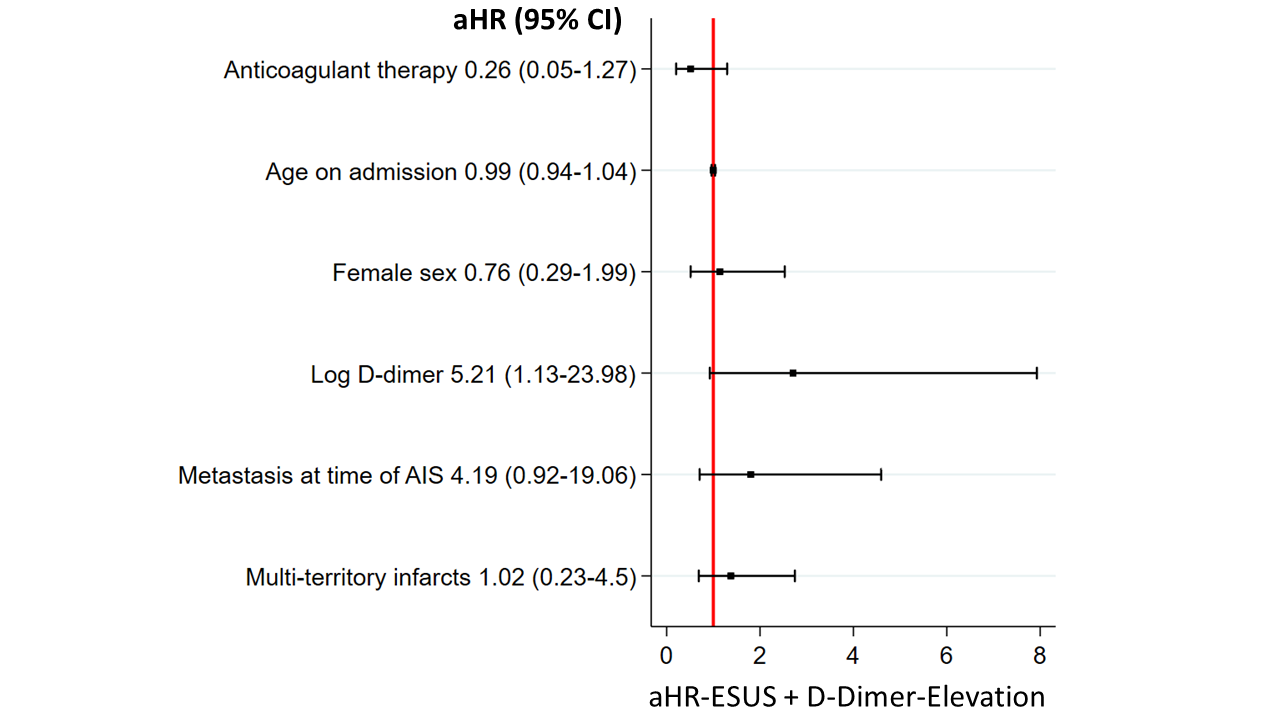


**eFigure VI –** Multivariable model studying the association between anticoagulant versus antiplatelet therapy and 1-year mortality in the subgroup of patients with ESUS and elevated D-dimer levels.

An elevated D-dimer was defined as a value above the median (2,037 µg/L). There was no statistically significant association between anticoagulant therapy and mortality after adjustment for potential confounders. D-dimer was log-transformed due to its skewed distribution.

Abbreviations: aHR, adjusted hazard ratio; AIS, acute ischemic stroke.


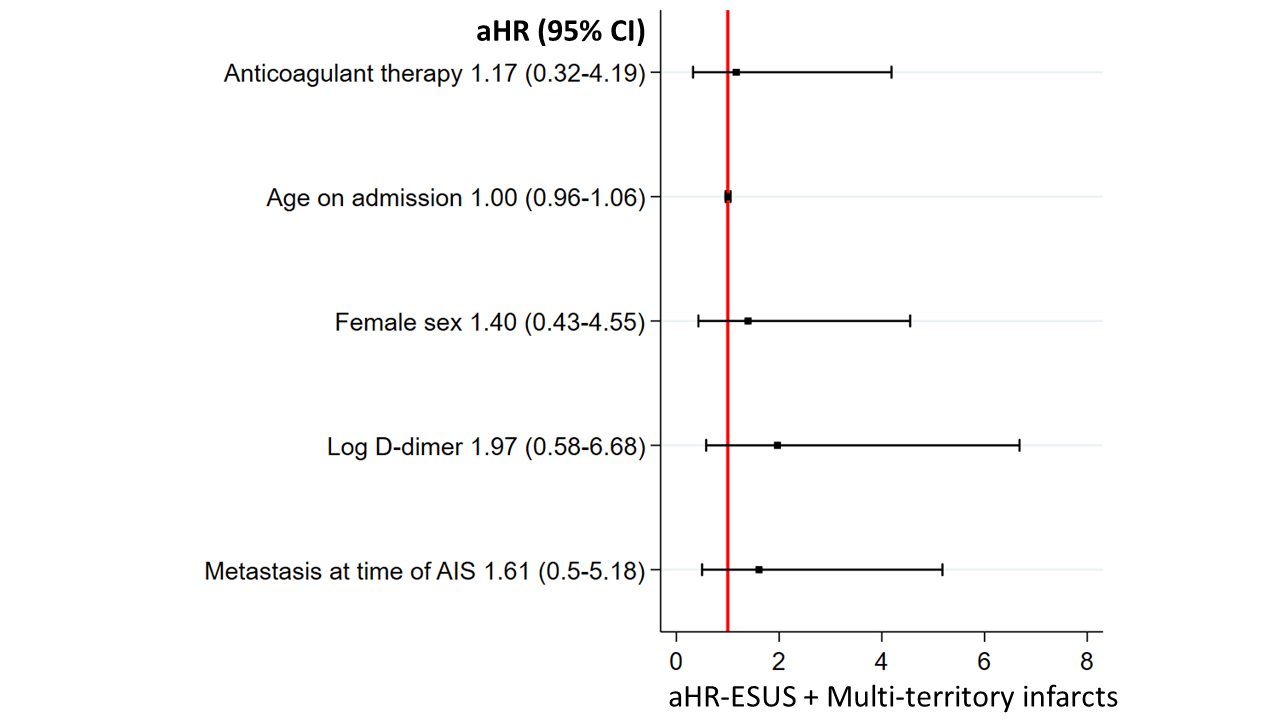


**eFigure VII –** Multivariable model studying the association between anticoagulant versus antiplatelet therapy and 1-year mortality in the subgroup of patients with ESUS and multiterritory infarcts.

No statistically significant association was observed between treatment strategy and mortality after adjustment for potential confounders. D-dimer was log-transformed due to its skewed distribution.

Abbreviations: aHR, adjusted hazard ratio; AIS, acute ischemic stroke.


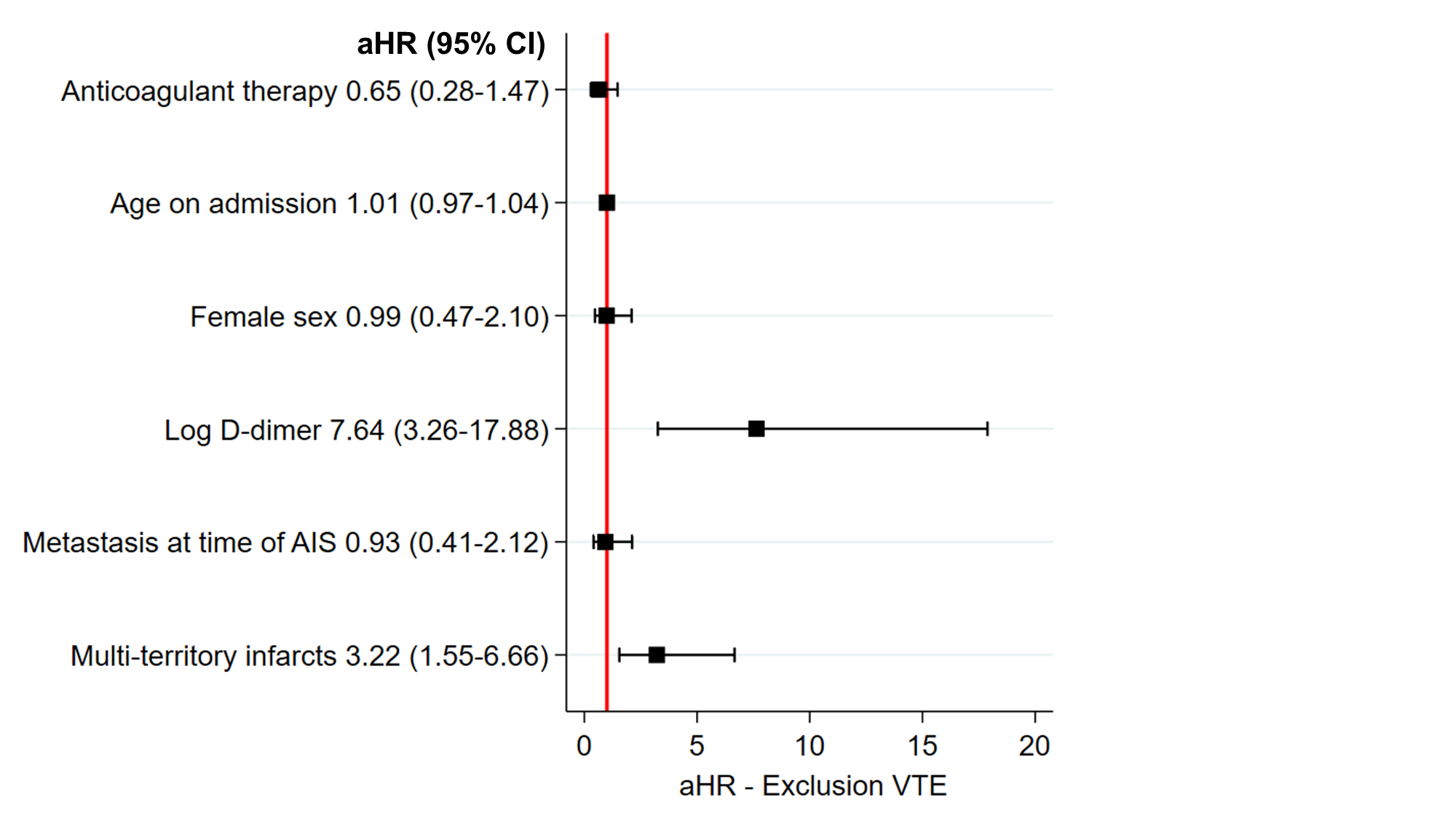


**eFigure VIII** – Multivariable model studying the association between antithrombotic treatment strategies at hospital discharge for AIS and 1-year mortality after excluding patients with VTE prescribed anticoagulant therapy at discharge.

There was no association between anticoagulant therapy, as compared to antiplatelet therapy, and 1-year mortality in this analysis. D-dimer was abnormally distributed so it was log transformed. Abbreviations: aHR, adjusted hazard ratio; AIS, acute ischemic stroke and VTE, venous thromboembolism.

**eFigure IX** – Multivariable model studying the association between antithrombotic treatment strategies at hospital discharge for AIS and 1-year mortality in the ESUS subgroup after excluding patients with VTE prescribed anticoagulant therapy at discharge.

There was no association between anticoagulant therapy, as compared to antiplatelet therapy, and 1-year mortality in this analysis. D-dimer was abnormally distributed so it was log transformed. Abbreviations: aHR, adjusted hazard ratio; AIS, acute ischemic stroke; ESUS, embolic stroke of undetermined source and VTE, venous thromboembolism.


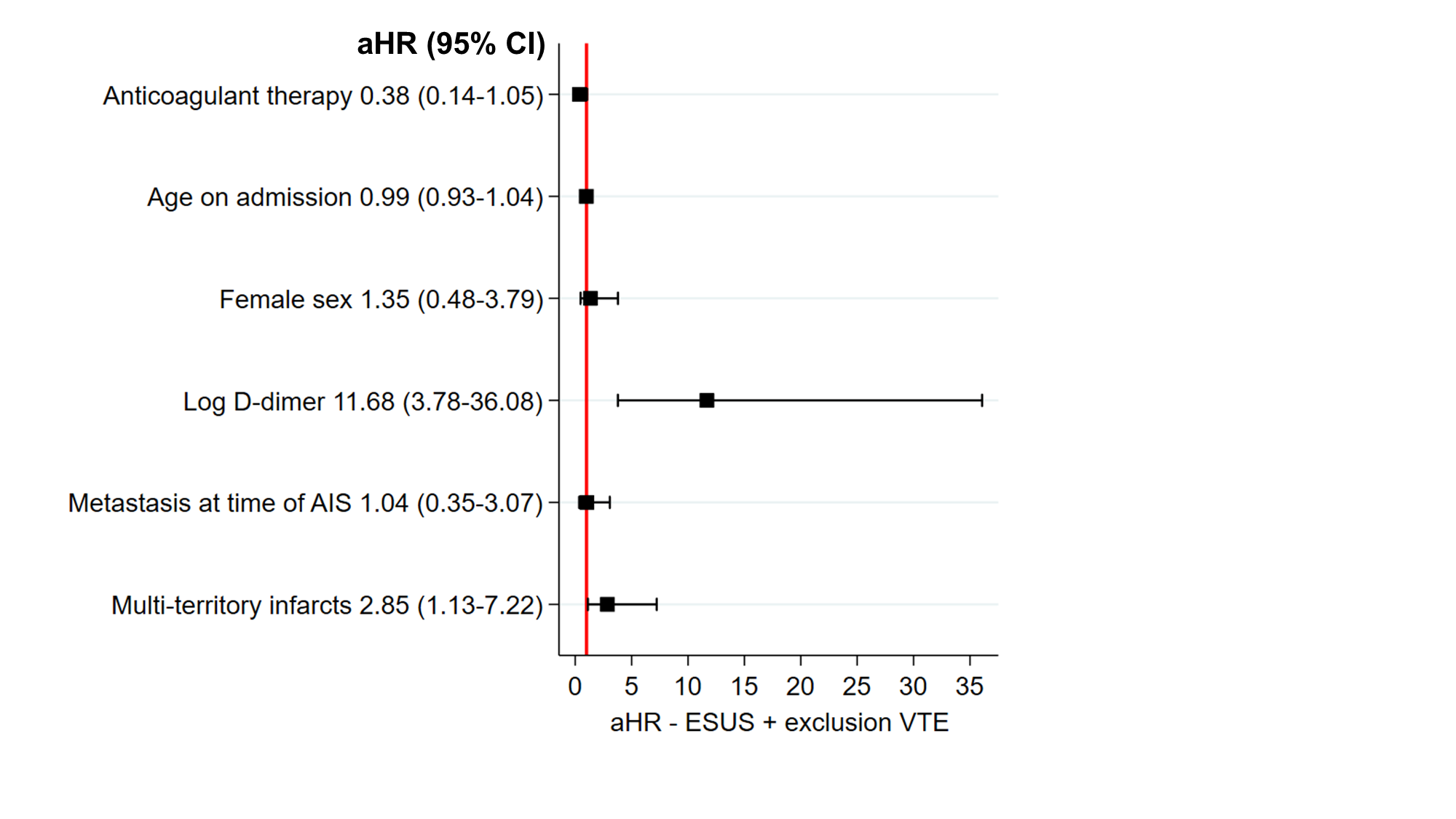

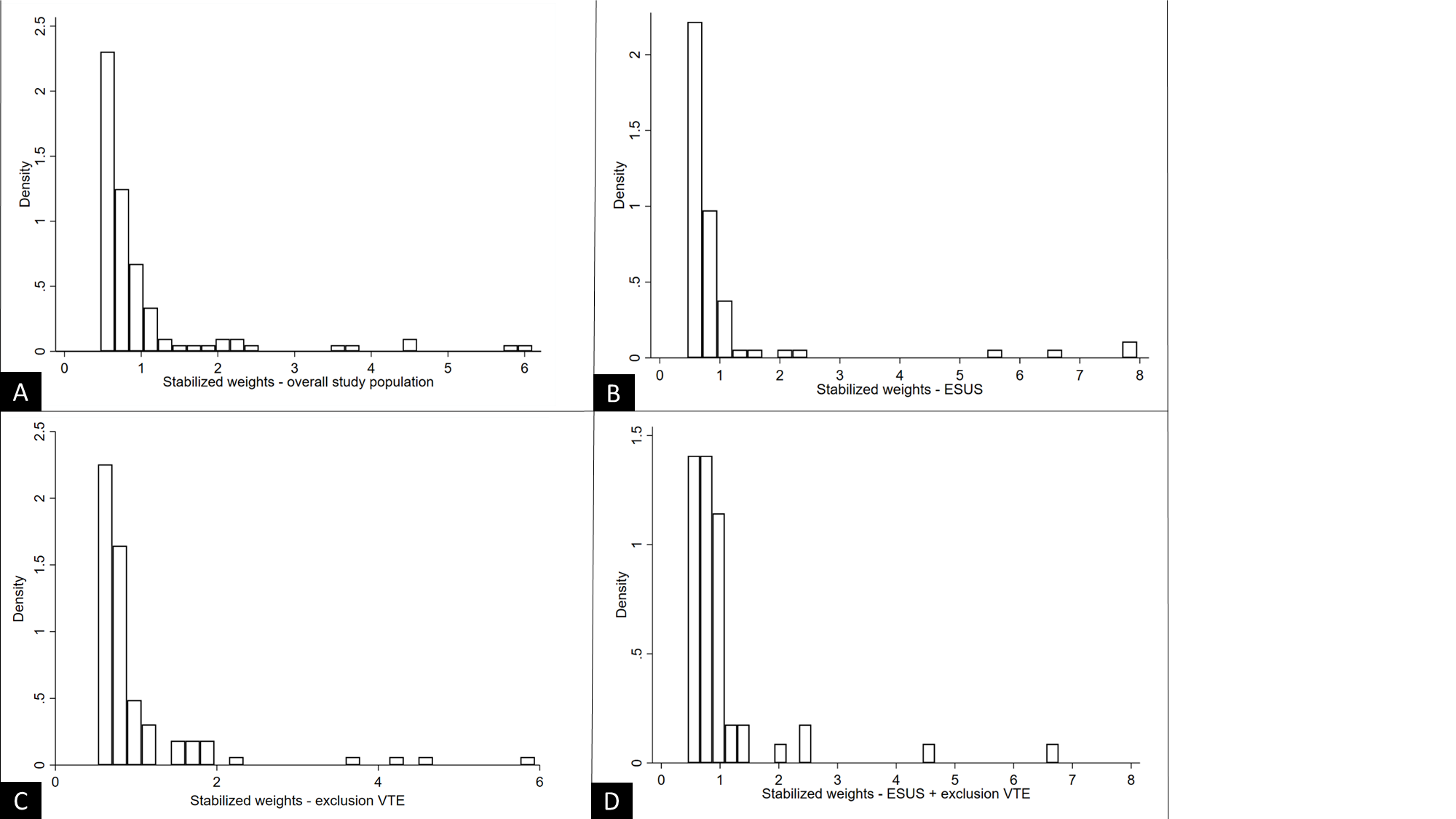


**eFigure X** – Stabilized weights analysis in the analyses employing IPTW.

Assessment of the stabilized weights for the mentioned patient groups (A-D) with distribution close to normal centered around one, albeit with a few weights surpassing two, potentially exerting a considerable influence on the outcomes in all groups. Abbreviations: ESUS, embolic stroke of undetermined source, IPTW, inverse probability of treatment weighting and VTE, venous thromboembolism.


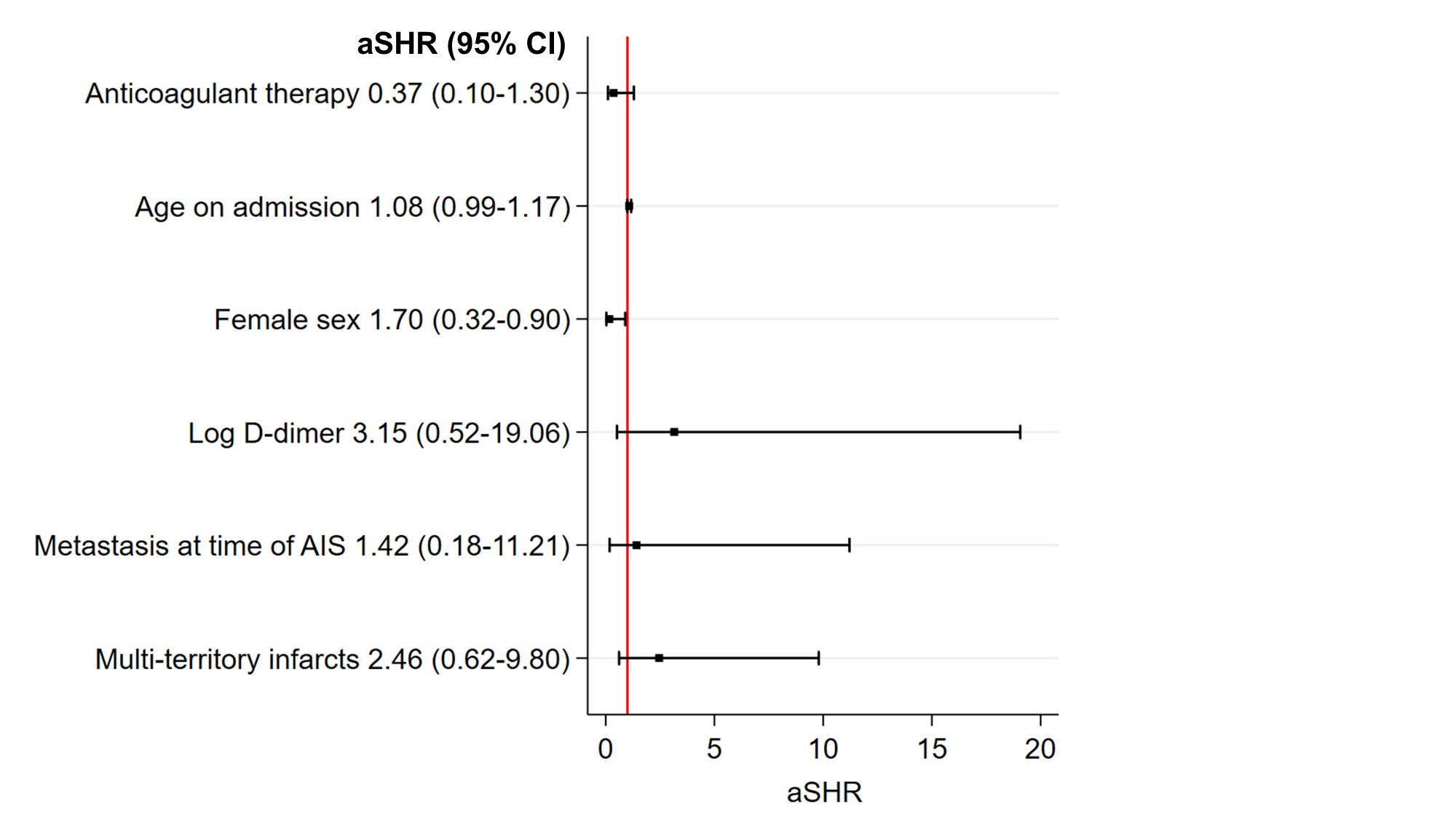


**eFigure XI –** Multivariable model studying the association between antithrombotic treatment strategies at hospital discharge for AIS and recurrent AIS during long-term follow-up with mortality as competing risk.

When accounting for mortality as a competing risk, there was no association between anticoagulant therapy, as compared to antiplatelet therapy, and recurrent AIS during long-term follow-up in this analysis.

D-dimer was abnormally distributed so it was log transformed. Abbreviations: aSHR, adjusted Subhazard ratio and AIS, acute ischemic stroke.
